# A Microarray Platform-Independent Classification Tool for Cell of Origin Class Allows Comparative Analysis of Gene Expression in Diffuse Large B-cell Lymphoma

**DOI:** 10.1371/journal.pone.0055895

**Published:** 2013-02-12

**Authors:** Matthew A. Care, Sharon Barrans, Lisa Worrillow, Andrew Jack, David R. Westhead, Reuben M. Tooze

**Affiliations:** 1 Section of Experimental Haematology, Leeds Institute of Molecular Medicine, University of Leeds, Leeds, United Kingdom; 2 Bioinformatics Group, Institute of Molecular and Cellular Biology, University of Leeds, Leeds, United Kingdom; 3 Haematological Malignancy Diagnostic Service (HMDS), St. James’s Institute of Oncology, Leeds, United Kingdom; University of North Carolina at Chapel Hill, United States of America

## Abstract

Cell of origin classification of diffuse large B-cell lymphoma (DLBCL) identifies subsets with biological and clinical significance. Despite the established nature of the classification existing studies display variability in classifier implementation, and a comparative analysis across multiple data sets is lacking. Here we describe the validation of a cell of origin classifier for DLBCL, based on balanced voting between 4 machine-learning tools: the DLBCL automatic classifier (DAC). This shows superior survival separation for assigned Activated B-cell (ABC) and Germinal Center B-cell (GCB) DLBCL classes relative to a range of other classifiers. DAC is effective on data derived from multiple microarray platforms and formalin fixed paraffin embedded samples and is parsimonious, using 20 classifier genes. We use DAC to perform a comparative analysis of gene expression in 10 data sets (2030 cases). We generate ranked meta-profiles of genes showing consistent class-association using ≥6 data sets as a cut-off: ABC (414 genes) and GCB (415 genes). The transcription factor *ZBTB32* emerges as the most consistent and differentially expressed gene in ABC-DLBCL while other transcription factors such as *ARID3A*, *BATF,* and *TCF4* are also amongst the 24 genes associated with this class in all datasets. Analysis of enrichment of 12323 gene signatures against meta-profiles and all data sets individually confirms consistent associations with signatures of molecular pathways, chromosomal cytobands, and transcription factor binding sites. We provide DAC as an open access Windows application, and the accompanying meta-analyses as a resource.

## Introduction

Diffuse large B-cell lymphomas (DLBCL), the commonest human lymphoma type, can be separated into distinct categories based on gene expression signature, and relationship to normal stages of B-cell differentiation [1], [2], [3]. The success of the “cell of origin” classification lies in the ability to both predict differences in patient outcome with standard immuno-chemotherapy regimens, and provide insight into the fundamental biology of the disease [4], [5]. An extensive body of work has linked the two major categories of this classification, the Germinal Center B-cell (GCB) and Activated B-cell (ABC) types of DLBCL, to different molecular pathogenesis [6]. Further validation of this paradigm has been provided recently in the context of global analysis of coding region mutations in DLBCL, which link different spectra of somatic mutations to cell of origin class [7], [8].

Since the inception of the cell of origin classification [1], several variations have been used. The definitive formulation, by Wright et al., employed 27-features present on the Lymphochip custom array to assign cases as ABC, GCB or Type-III/unclassified [2]. Classes were assigned using a linear predictive score (LPS) derived from the expression values of these features, with the resulting score for each case assessed using a Bayesian predictor against training data set distributions. ABC or GCB class was assigned where the respective class prediction was over 90% certain, with cases falling between these extremes assigned to the unclassified (also known as Type-III) category. Application in subsequent work has seen the gradual expansion of the classifier gene set; a process that accompanied extension to encompass the distinction of DLBCL from Burkitt Lymphoma and Primary Mediastinal B-cell Lymphoma by Dave et al. [9] (a 2-stage classifier, the 2^nd^ stage using 100 genes to differentiate Burkitt Lymphoma from each subgroup of DLBCL), and the application to patients treated with chemotherapy regimes supplemented by anti-CD20 monoclonal antibody therapy by Lenz et al. [5] (183 genes). In parallel other studies have applied the original Wright algorithm in a “truncated” form to reflect the variable representation of classifier genes on more recent platforms. This is exemplified in the work of Monti et al. (23 genes), who identified DLBCL subtypes characterised by B-cell receptor, oxidative phosphorylation and host response signatures [10], and Hummel et al. [11] (15 genes) describing the molecular identification of Burkitt lymphoma concurrent with Dave et al. [9]. Despite the variation in classifier gene number, a unifying feature of these, and other studies [12], [13], [14], has been the use of a Bayesian predictor as described by Wright et al. and extended in subsequent work [2], [5], [9]. However no published studies share an identical approach to classification with variations in classifier gene number or precise detail of classifier implementation such as the way in which probes for individual genes are selected or normalized.

In routine practice most diagnostic material is formalin-fixed and paraffin embedded (FFPE), which may not yield comparable gene expression data to that obtained from fresh material. While approaches have been developed to circumvent this issue, immunohistochemical surrogates fail to recapitulate the success of the gene expression based classifier on a consistent basis [15], [16]. Targeted assessments of individual genes by quantitative PCR [17], [18] or by RNAse protection assays [19], [20] have been shown to be effective as classifier tools. However the initial selection of target genes imposes inherent restrictions on downstream analysis and development of new classifiers in the context for example of clinical trials, which is not the case in the context of global gene expression analysis on microarray platforms. FFPE based gene expression profiling of DLBCL has now been tested in several experimental settings [21], [22], [23], including the demonstration that FFPE samples processed entirely as conventional diagnostic material can provide meaningful data for prediction of survival [22].

The segregation of DLBCL into cell of origin classes has led to profound insight into disease biology [6]. This is reflected in the fact that extended patterns of gene expression associated with cell of origin class link to underlying molecular abnormalities. When considering an individual data set the primary criteria for ranking the association of a gene with disease class are the significance and magnitude of differential expression. Comparison of multiple equivalently classified data sets can generate an important additional variable: the consistency with which a gene is associated with disease class across the data sets considered. Determining such consistency of association provides an approach for comparative analysis of gene expression that avoids issues related to merging expression values from different data sets prior to classification. This approach depends on a classifier implementation that is robust to variation between microarray platforms and data sources. Multiple individual data sets of DLBCL have now been generated [5], [10], [11], [12], [21], [23], [24], [25], [26], providing the potential basis for such a comparative analysis. In parallel extensive databases have been established encompassing thousands of gene lists (referred to as gene signatures or gene sets) that identify differentially expressed genes from prior microarray experiments from a wide array of cell types and conditions [27], [28]. These include lists of genes defined by other parameters such as chromosomal distribution (cytobands) or association with conserved transcription factor binding sites in promoter regions and 3′UTRs [29]. Combining a comparative analysis of gene expression with a comprehensive assessment of gene signature enrichment generates a resource and provides a tool for assessing classifier performance.

Here we describe a systematic analysis of tools for implementation of the cell of origin classification of DLBCL. We establish a platform robust classifier based on balanced voting between four machine-learning tools. This is effective on FFPE material, and provides improved survival separation for ABC and GCB classes for the majority of data sets analyzed. We make this tool available as open source software. The development of this tool allows the first comparative analysis of gene expression across 10 DLBCL data sets encompassing 2030 cases uniformly classified with the same implementation. We define meta-profiles of genes consistently associated with ABC- and GCB class, assess consistent molecular signature enrichments and provide these data as a resource.

## Materials and Methods

### Datasets

A formalin-fixed paraffin embedded (FFPE) data set was produced by the Haematological Malignancy Diagnostic Service (HMDS; St. James’s Institute of Oncology, Leeds) and details of preparation, epidemiology and outcome data are described in detail elsewhere [22]. The data is available from the NCBI Gene Expression Omnibus (GEO: GSE32918). For training the machine learning tools the Lymphochip-based data set of Wright et al. was downloaded (http://llmpp.nih.gov/DLBCLpredictor/) [2]. In addition 9 diffuse large B-cell lymphoma (DLBCL) datasets were downloaded from the GEO and elsewhere: GSE10846, GSE12195, GSE19246, GSE22470, GSE22895, GSE31312, GSE34171, GSE4475, and the data of Monti et al. (Table S1) [5], [10], [11], [12], [21], [23], [24], [25], [26]. The data set, GSE10846, was split into treatment groups (CHOP/R-CHOP) yielding two data sets that were then analyzed independently (referred to as GSE10846_CHOP and GSE10846_R-CHOP) thus giving a total of 11 data sets.

### Normalization and Re-annotation of Data

Each dataset was quantile normalized using the R Limma package [30]. To maximise integration of data, spanning different resources and from different sources (e.g. expression arrays, gene ontology annotations, gene lists etc), all genes across all used resources were re-annotated to HUGO Gene Nomenclature Committee (HGNC) approved symbols. The complete HGNC list was downloaded (on 2012.08.16). Each gene was re-annotated to the latest approved symbol if an unambiguous mapping (i.e. single symbol mapping to an approved symbol) could be determined, else the original gene name was maintained.

### DLBCL Classifier Generation: Data Preparation

For all data sets the probes for each of the classifier genes were merged using 2 methods: (1) the median value across the probes for each gene (**MedianMerge**), and (2) the approach used by Monti et al. - taking the average value for probes with a Pearson correlation >0.2, while taking the maximum value for those with a correlation < = 0.2 (**MaxAvgMerge**) [10]. The median approach proved to be more informative for all but one data set (data not shown) and thus was used unless stated. The resulting values were used to generate Z-scores for each classifier gene in a particular data set. These values were then converted to ARFF files for use with the machine learning library Weka [31].

### DLBCL Classifier Generation: Classifier Ranking

The Survival library for R was used to analyze right-censored survival data, overall survival was estimated using the Kaplan-Meier method, modeled with Cox Proportional Hazards technique [32]. There proved to be poor correlation between a classifiers cross-validation score and the separation of its classes in survival curves, with many classifiers having optimistic cross-validations scores (Table S3 and Table 1). Thus classifiers were ranked only according to separation of survival (for ABC/GCB).

**Table 1:** Assessment of individual machine-learning tool classifiers.

		HMDS (GSE32918)				GSE10846 R-CHOP				GSE10846 CHOP			
Classifier	Average Rank	Hazard Ratio	95% Conf Intervals		*P*-Val	Hazard Ratio	95% Conf Intervals		*P*-Val	Hazard Ratio	95% Conf Intervals		*P*-Val
SMO	3.7	0.56	0.32	1.01	0.052	0.25	0.14	0.46	8.07E-06	0.36	0.23	0.55	2.06E-06
LMT	12.0	0.57	0.33	0.98	0.043	0.28	0.15	0.50	2.35E-05	0.40	0.26	0.61	3.17E-05
BayesNet	14.3	0.67	0.38	1.17	0.155	0.24	0.13	0.45	9.18E-06	0.41	0.26	0.64	7.47E-05
J48	15.0	0.68	0.40	1.17	0.162	0.28	0.15	0.50	1.58E-05	0.41	0.27	0.63	4.29E-05
RF100	16.3	0.85	0.48	1.48	0.561	0.24	0.13	0.44	3.34E-06	0.41	0.27	0.63	5.06E-05
RF200	18.3	0.78	0.45	1.35	0.379	0.24	0.13	0.44	3.79E-06	0.44	0.29	0.68	1.81E-04
LPS 0.9 MaxAvgMerge	21.3	0.68	0.36	1.27	0.226	0.31	0.17	0.55	8.49E-05	0.40	0.26	0.63	7.65E-05
LPS 0.9 MedianMerge	21.7	0.64	0.34	1.20	0.162	0.32	0.17	0.58	1.77E-04	0.42	0.27	0.65	1.19E-04
LPS 0.8 MedianMerge	22.3	0.73	0.41	1.28	0.267	0.31	0.17	0.54	5.60E-05	0.44	0.29	0.67	1.17E-04
LPS 0.8 MaxAvgMerge	23.7	0.71	0.40	1.25	0.235	0.33	0.19	0.58	1.04E-04	0.43	0.28	0.66	1.20E-04
REPTree	24.7	1.51	0.85	2.69	0.164	0.43	0.24	0.77	4.64E-03	0.47	0.31	0.71	4.03E-04
FT	27.7	0.77	0.41	1.46	0.426	0.33	0.18	0.61	3.92E-04	0.48	0.30	0.76	1.92E-03
BFTree	28.7	0.91	0.51	1.64	0.758	0.40	0.22	0.72	2.33E-03	0.50	0.32	0.76	1.38E-03
NBTree	28.7	0.96	0.50	1.88	0.915	0.29	0.15	0.52	5.61E-05	0.57	0.37	0.89	1.30E-02
RandomTree	28.7	0.97	0.55	1.72	0.914	0.27	0.13	0.57	5.58E-04	0.44	0.28	0.70	4.97E-04
SimpleCart	30.3	0.93	0.52	1.67	0.804	0.48	0.27	0.85	1.27E-02	0.56	0.36	0.86	8.46E-03

Results obtained with individual machine-learning tools, trained on the Wright et al. data set and using 20 classifier genes are shown. Survival separation between ABC and GCB classes for the data sets GSE32918, and GSE10846 divided into CHOP and R-CHOP components, was used for assessment. Hazard Ratios were generated for GCB relative to ABC as baseline. The classifiers were ordered by their average rank across the data sets; with rank determined by the p-value of the ABC/GCB separation. The LPS classifier was used for comparison with either a 0.8 or 0.9 p-value cut-off, with either MaxAvgMerge or MedianMerge methods of combining probes (see Materials and Methods). The Classifier Identity, Hazard Ratio (GCB vs ABC as baseline), 95% confidence interval of the Hazard Ratio, and the resulting p-value for survival separation are shown.

### DLBCL Classifier Generation: Linear Predictor Score (LPS) Classifier

An implementation of the LPS classifier was generated that can process the Weka ARFF file format [2]. This allowed a direct comparison of an existing cell of origin classifier against the Weka machine learning tools.

### DLBCL Classifier Generation: Classifiers (Figure 1: step 1 and 2)

Using the Wright et al data as training set, 12 machine learning tools (BayesNet, BFTree, FT, J48, LMT, NBTree, RandomTree, REPTree, RF100, RF200, SimpleCart and SMO) were trained using the Weka package (Weka version 3.6.5) [31]. For all tools default settings were used except the random forests (RF100/RF200), which were set to 100/200 trees respectively. In addition, 4 different LPS classifiers were “trained” on the Wright data (LPS0.9MaxAvgMerge, LPS0.9MedianMerge, LPS0.8MedianMerge and LPS0.8MaxAvgMerge; relating to the LPS p-value cut-off (0.8/0.9) and the method of merging probes for classifier genes (MedianMerge/MaxAvgMerge see Data preparation)).

The Wright et al. data set consists of expression data for 240 patients annotated as one of three classes: ABC (n = 73; 30.4%), GCB (n = 115; 47.9%) or Type-III (n = 52; 21.7%). The trained Weka classifiers output predictions for each sample analyzed consisting of p-values for each of the 3 classes, the class with the largest p-value giving the predicted class. The LPS classifiers assign everything with a confidence greater than a p-value threshold (0.8/0.9) to ABC/GCB and everything below the threshold to Type-III/Unclassified. The predicted classes were used to rank the classifiers (see Classifier Ranking).

The 6 best individual Weka classifiers (LMT, SMO, BayesNet, J48, RF100, RF200) were combined using the Weka Vote scheme with the average of probabilities combination rule. Each classifier was removed in turn to generate all possible 5-tool classifiers (5N). This process was iterated with the best meta-classifier at each N progressing to the next level (e.g. best 5N → all possible 4N). As with the individual classifiers these were analyzed by comparing the survival of their ABC/GCB assigned cases.

### DLBCL Classifier Generation: Generation of Classifier Gene Sets (Figure 1: step 3)

The data sets GSE10846 (split into CHOP/R-CHOP), GSE4475 and GSE19246 were used to generate additional lists of classifier genes from 3 sources using the published classifications (ABC, GCB and Type-III/unclassified): (1) the 20 Wright genes were ranked using the Weka CfsSubsetEval method (search method: GreedyStepwise). (2) the 185 genes from Dave et al. present on the 3 platforms were ranked using CfsSubsetEval (search method: GreedyStepwise) [9]. (3) all genes on each platform were ranked using the Weka InfoGainAttributeEval (search method: Ranker). The top 1000 from each data set were then ranked again using the more computationally expensive CfsSubsetEval method. In order to find the most consistently informative genes across all 4 data sets, the final rank was generated by averaging the ranks across the data sets. Using these ranks the following lists were generated: Wright 5 and 10; Dave 10, 20, 50, 100 and 185; All 10, 20, 50, 100 and 185 (Table S2).

### Identification of Differentially Expressed Genes (Figure 1: step 5)

Using the classes assigned by the best classifier (LMT_J48_RF100_SMO) a linear model was fitted to the gene expression data using the R Limma package [30]. Differentially expressed genes between the classes were gauged using the Limma empirical Bayes statistics module, adjusting for multiple testing using Benjamini & Hochberg correction. These lists of differentially expressed genes were then used for downstream analysis.

### Enrichment Analysis

Enrichment of genes against gene-lists was assessed using a hypergeometric test, where the draw is the significantly differentially expressed genes, the successes are the signature genes and the population is the genes present on the platform. To avoid any bias the genes used for training the machine-learning tool were removed from the signatures before assessment. For each assessment Z-scores were generated by comparing against random distributions.

### Comparison of Dataset Classes

The ABC/GCB classification of the 11 DLBCL datasets (GSE10846 split in 2) was assessed by comparing the overlap between the up-regulated genes in ABC/GCB for each dataset against all others. The genes that were significantly up-regulated (adjusted p-value <0.05) in ABC/GCB were defined in each data set, creating a set of gene lists linked to either ABC or GCB class. Enrichment analysis (described above) was then carried out for differentially expressed genes from each dataset individually against the set of gene lists defined for all other data sets for each class (e.g. ABC genes data set 1 vs ABC genes data set 2, ABC genes data set 1 vs ABC genes data set 3 etc.) (Z-scores from random distributions of 10^7^ samples). The Z-scores were then averaged between the two directions of analysis (data set 1 vs data set 2 and data set 2 vs data set 1) and also between ABC/GCB.

### Enrichment of Gene Sets

A data set of 12,323 gene signatures was created by merging signatures downloaded from http://Lymphochip.nih.gov/signaturedb/(SignatureDB), http://www.broadinstitute.org/gsea/msigdb/index.jsp MSigDB V3.1 (**MSigDB C1–C6**), http://compbio.dfci.harvard.edu/genesigdb/Gene Signature Datbase V4 (**GeneSigDB**) and 3 individual papers [10], [27], [33], [34], [35], [36].

Signature enrichment analysis was carried out for the significantly differentially expressed genes (adjusted p-value <0.05). The contrast of ABC and GCB classes yields two lists, those up-regulated in ABC or GCB, which were analyzed separately. Enrichment of the signatures was assessed as described above.

### Figures

Heat-maps were generated using the MEV program from the TM4 package of microarray tools [37], and chromosome regions were drawn using the R libraries ggbio and ggplot2 [38], [39].

### Implementation of Automatic Classifier

A graphical user interface (GUI) driven implementation of the DAC was created to simplify the classification process for users. The code behind the GUI was written in Python (http://www.python.org/) while the GUI was generated using the WxPython (http://www.wxpython.org/) toolkit.

The classifier takes as input a tab separated list of raw gene/probe expression values. The file is quantile normalised (normalizeQuantiles function of R Limma package) and then if there are multiple probes for a gene these are merged by taking their median value. Finally, Z-scores are generated for each gene across the samples [30]. The Z-scores for the 20 classifier genes (or as many as exist on the platform) are used to produce an attribute-relation file format (ARFF) file for analysis by Weka [31]. A Weka classifier is generated using the Wright et al. data set with the same subset of classifier genes. Finally, the resulting Weka output is post-processed to append sample names and tidy up the format for easier analysis.

The automatic classifier allows a background file (>30 samples of random class generated on the equivalent platform) to be used for classification of individual samples. The program finds the genes shared between the two files, though ideally the two files should be from the exact same platform. The file to be classified (1 or more samples) is split into individual samples and each of these is separately appended to the background file, followed by quantile normalization and generation of Z-scores. Once this process finishes for all individual samples the resulting Z-scores are merged and used to generate an ARFF file, which is then processed as above.

The classifier and manual is available from: http://www.bioinformatics.leeds.ac.uk/~bgy7mc/DAC/.

## Results

### Implementation of a Transferable Classifier: Overview

Our goal was to establish an implementation of the cell of origin classifier that was robust against variation in microarray platform and fresh or FFPE sample type. The cell of origin classifier is distinguished by two linked characteristics: (1) the ability to define two primary classes of DLBCL, ABC and GCB, with significant differences in outcome when treated with the combination chemotherapy regimen CHOP, (cyclophosphamide, hydroxydaunorubicin, vincristine (Oncovin), and prednisolone), alone or including rituximab anti-CD20 monoclonal antibody therapy (R-CHOP), and (2) the fact that these classes are linked to extended patterns of gene expression reflecting underlying molecular pathogenesis. Our assessment of classifier performance was therefore based first on the ability to define ABC and GCB classes with differences in outcome, using overall survival, and second on the demonstration that the defined classes of DLBCL across multiple data sets showed similar overall patterns of gene expression and appropriate segregation of non-classifier genes and molecular signatures.

In addition to the data set generated on a custom Lymphochip spotted cDNA microarray, on which the original formulation of the cell of origin classification by Wright et al. was based [2] (referred to as Wright et al. data), we identified 9 additional data sets from the literature and the Gene Expression Omnibus (GEO, referred to by Gene Expression Omnibus accession number where applicable, Table S1): GSE10846, GSE12195, GSE19246, GSE22470, GSE22895, GSE31312, GSE34171, GSE4475, and the data of Monti et al. [5], [10], [11], [12], [21], [23], [24], [25], [26]. A batch effect is evident in the largest data set with available outcome data, GSE10846, between samples derived from CHOP and R-CHOP treatment, and these two parts of the data set were therefore treated separately. In addition we generated a data set in-house, GSE32918, from FFPE material derived from diagnostic biopsies of a population-based cohort treated with R-CHOP; the epidemiology, treatment and outcome data of which are described elsewhere [22].

**Figure 1:**
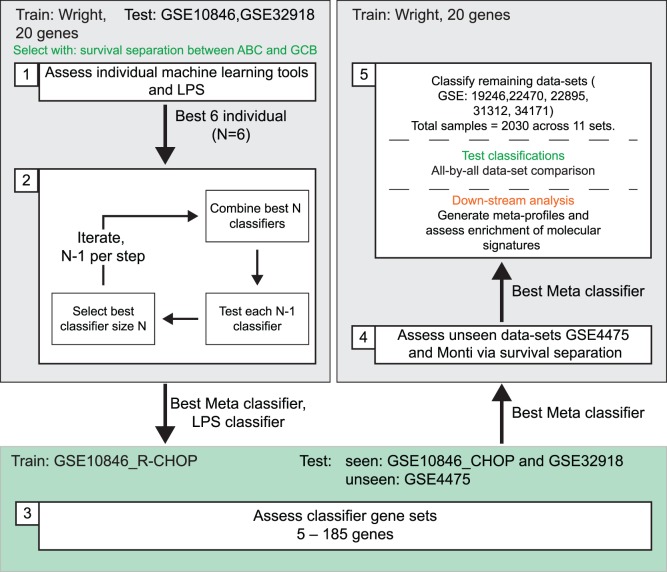
Overview of classifier generation, testing and downstream analysis. Colored boxes (gray/green) depict different training data sets. Step 1- assessment of individual machine-learning tools vs LPS; Step 2– assessment of machine-learning tool combinations; Step 3–assessment of classifier gene sets, training on GSE10846_R-CHOP, and testing on previously seen and unseen data sets: Step 4- further assessment on unseen data sets; Step 5– classification of additional data sets, evaluation of differential gene expression in all-by-all comparison, downstream analysis with meta-profiles and enrichment of molecular signatures.

We used two data sets (GSE32918, and GSE10846 divided into CHOP and R-CHOP treated groups) for classifier development. At each stage classifiers were ranked by the survival separation between the assigned ABC/GCB classes (see Classifier ranking in methods). We proceeded through the following steps (Figure 1): (1) we assessed the performance of 12 individual machine-learning classifiers, trained on the Wright et al. data and using 20 Wright classifier genes, the same data was analyzed using an in-house implementation of the LPS classifier [2], and results were compared to the GEO published classes; (2) we assessed the value of combining tools starting with the best 6 and removing each tool individually, finding the best combination of 5 classifiers, and again removing each remaining tool, and performing this cycle iteratively; (3) we assessed the impact of training on an alternate data set and different combinations of classifier genes (n = 5−185) using the best meta-classifier (from step 2) or the LPS classifier of Wright et al. [2]; (4) the resulting selected classifier was then assessed using two additional, unseen, data sets with sufficient samples and linked outcome data (GSE4475 and Monti et al.), as these data sets were not used to generate the meta-classifier they provide an unbiased assessment of performance [10], [11]; (5) the remaining data sets were classified and used to assess appropriate segregation of gene expression with class across all 10 data sets, representing 2030 samples. Finally the equivalently classified data sets were used to establish meta-profiles and assess enrichment of molecular signatures.

### Machine-learning Tool Comparison

Twenty of the 27 genes, described by Wright et al. [2], are represented both on the Illumina HumanRef-8 WG-DASL v3.0 and Affymetrix Human Genome U133 Plus 2.0 platforms used to generate GSE32918 and GSE10846 respectively (3 Refseq genes used by Wright et al. are not included on the Illumina platform, and the remaining 4 features have not been confirmed as Refseq genes and are not present on either platform) (Table S2). We first tested the ability of machine-learning tools to use these 20 genes to generate cell of origin classifiers, using the Wright et al. data as a training set, by 10-fold cross-validation (Table S3). We then compared survival separations, using overall survival, for the ABC and GCB classes generated by these classifiers in the data sets GSE32918 (FFPE) and GSE10846 (fresh frozen) divided into CHOP and R-CHOP subsets. The LPS classifier defined prognostic groups in the GSE10846 data sets (R-CHOP component, GCB vs ABC Hazard Ratio = 0.31, p-value = 8.49E-05), but failed to define groups with significant difference in survival in the FFPE derived data GSE32918 (GCB vs ABC Hazard Ratio = 0.64, p-value = 0.1618). In both data sets the LPS classifier was outperformed for survival separation by several machine-learning tools (Table 1 and Table S3). For GSE10846 published assignments generated by a classifier using 183 genes, were also available. While these classification choices (GEO Class) provided better survival separation than those made by LPS (e.g. R-CHOP component, GCB vs ABC Hazard Ratio = 0.297, p-value = 4.42E-05), they generated less significant survival separation than classification choices made by several of the individual machine-learning tools (e.g. R-CHOP component RF100 classifier, GCB vs ABC Hazard Ratio = 0.238, p-value = 3.34E-06).

### Machine-learning Tool Combinations

Machine-learning tools can be combined to provide the potential advantage of balanced voting between classifiers generated from individual tools. We again used survival separation between assigned ABC and GCB classes as a metric to test the performance of balanced voting between classifiers. To assess such meta-classifiers we combined the best 6 individual machine-learning tools (Table S3) (LMT, SMO, BayesNet, J48, RF100, RF200) then removed each contributing tool individually and selected the best 5-tool classifier, then repeated the cycle iteratively to arrive at 4-tool, 3-tool and 2-tool meta-classifiers. While balanced voting by 6 machine-learning tools did not improve outcome separation, balanced voting by fewer classifiers did improve survival separation of classes relative to most individual machine-learning tools (Table 2 **)**.

**Table 2:** Assessment of machine-learning tool meta-classifiers combined with balanced voting.

		HMDS (GSE32918)				GSE10846 R-CHOP				GSE10846 CHOP			
Classifier	Average Rank	Hazard Ratio	95% Conf Intervals		*P*-Val	Hazard Ratio	95% Conf Intervals		*P*-Val	Hazard Ratio	95% Conf Intervals		*P*-Val
LMT_J48_RF100_SMO	5.0	0.56	0.33	0.97	0.037	0.26	0.14	0.47	9.88E-06	0.37	0.24	0.57	6.38E-06
LMT_RF200_RF100_SMO	5.7	0.65	0.37	1.15	0.136	0.24	0.13	0.43	3.16E-06	0.36	0.23	0.55	2.21E-06
RF100_SMO	5.7	0.58	0.33	1.02	0.060	0.26	0.14	0.47	8.66E-06	0.37	0.24	0.56	3.46E-06
LMT_RF100_SMO	6.7	0.65	0.37	1.13	0.126	0.25	0.14	0.45	4.88E-06	0.36	0.23	0.55	2.54E-06
J48_RF100_SMO	7.3	0.56	0.32	0.96	0.036	0.27	0.15	0.48	1.21E-05	0.40	0.26	0.61	2.21E-05
LMT_J48_RF200_SMO	7.7	0.59	0.34	1.01	0.054	0.26	0.14	0.47	9.88E-06	0.39	0.25	0.60	1.45E-05
J48_RF200_RF100_SMO	9.7	0.65	0.38	1.12	0.124	0.26	0.14	0.47	1.02E-05	0.39	0.25	0.59	1.43E-05
LMT_J48_RF200_RF100_SMO	10.0	0.62	0.36	1.07	0.085	0.26	0.14	0.47	9.88E-06	0.40	0.26	0.61	2.61E-05
LMT_J48_SMO	10.0	0.57	0.33	0.98	0.041	0.27	0.15	0.49	1.67E-05	0.40	0.26	0.62	3.09E-05
LMT_RF200_RF100_BN_SMO	12.0	0.64	0.37	1.11	0.112	0.29	0.16	0.53	5.43E-05	0.38	0.25	0.58	6.56E-06
J48_SMO	12.7	0.60	0.35	1.03	0.062	0.28	0.15	0.50	1.58E-05	0.41	0.27	0.63	4.29E-05
LMT_J48_RF200_RF100	12.7	0.60	0.35	1.02	0.061	0.26	0.14	0.48	1.38E-05	0.41	0.27	0.63	4.63E-05
LMT_J48_RF200_BN_SMO	13.0	0.72	0.41	1.24	0.230	0.26	0.14	0.48	1.35E-05	0.38	0.25	0.59	1.40E-05
LMT_J48_RF100_BN_SMO	14.0	0.72	0.41	1.24	0.230	0.26	0.14	0.48	1.35E-05	0.39	0.26	0.60	1.94E-05
LMT_J48_RF200_RF100_BN_SMO	15.7	0.72	0.41	1.24	0.230	0.26	0.14	0.48	1.35E-05	0.41	0.27	0.63	3.95E-05
LMT_J48_RF200_RF100_BN	17.7	0.70	0.41	1.22	0.211	0.27	0.15	0.49	1.95E-05	0.41	0.26	0.63	4.54E-05
J48_RF100	19.0	0.68	0.40	1.17	0.162	0.28	0.15	0.50	1.58E-05	0.46	0.30	0.70	2.95E-04
LMT_J48_RF100	19.0	0.70	0.41	1.20	0.193	0.27	0.15	0.49	1.78E-05	0.43	0.28	0.65	8.56E-05
J48_RF200_RF100_BN_SMO	19.3	0.74	0.43	1.28	0.284	0.27	0.15	0.49	1.71E-05	0.41	0.27	0.63	5.02E-05

Machine-learning tools were combined using balanced voting to generate meta-classifiers. The best 6 individual classifiers were combined, and with iterative cycles of classifier removal 5, 4, 3 and 2 machine-learning tool meta-classifiers were tested. Survival separation between assigned ABC and GCB classes for the data sets GSE32918, and GSE10846 divided into CHOP and R-CHOP components was used for assessment. The classifiers were ordered by their average rank across the data sets; with rank determined by the p-value of the ABC/GCB separation. The Classifier Identity, Hazard Ratio (vs ABC as baseline), 95% confidence interval of the Hazard Ratio, and the resulting p-value for survival separation are shown.

To arrive at a single classifier we considered both the rank of all classifiers assessed across the data sets and the percentage of cases assigned to ABC or GCB class, since improved segregation of outcome between ABC and GCB cases could come at the expense of fewer cases assigned to one or other of these classes, and increased assignment to Type-III/unclassified. Only three classifiers, LMT_J48_RF100_SMO, RF100_SMO, and SMO, were consistently ranked in the top 25% of all classifiers tested in every data set (8 out of 31 or better). Amongst these LMT_J48_RF100_SMO gave the lowest average assignment to the Type-III/unclassified subset (17%), and was therefore selected for further analysis (Table S4). This balanced voting classifier was also distinguished from SMO alone, by the ability to assign a wide range of classification confidences, a characteristic that proved to be of relevance in downstream analyses.

### Classifier Gene and Training Set Choices

A notable feature amongst published applications of the cell of origin classification is the variable number of genes used for classifier implementation (15 to 183) [5], [11]. This feature of the literature suggests that classifier gene number is not a critical determinant of performance. Furthermore machine-learning tools using 20 genes outperformed prior classification choices made using 183 genes in the separation of survival for the GSE10846 data set (Table S4). To explore this issue in more detail we directly assessed the performance of either LMT_J48_RF100_SMO or LPS using a range of different classifier gene numbers. Using the Weka CfsSubsetEval attribute selection method, we selected 10 and 5 classifier genes from the original 20 Wright genes (Wright20, Wright10, Wright5 in Table3). Similarly using the GEO classifications for GSE4475, GSE10846 and GSE19246 we selected the best genes, either from 185 derived from Dave et al. (Dave185 to Dave10 in Table 3) or using all present on an array (All185 to All10 in Table 3). The average rank across the data sets was used to give the most consistently informative classifier genes, generating sets of classifier genes of size: 185, 100, 50, 20 or 10.

**Table 3:** Effect of training data set and classifier gene number on survival separation.

	GSE10846 CHOP				GSE32918			
Classifier	Hazard Ratio	95% Conf Intervals		*P*-Val	Hazard Ratio	95% Conf Intervals		*P*-Val
GEO Published Class	0.41	0.27	0.64	6.43E-05	–	–	–	–
LMT_J48_RF100_SMO Classified								
Train Wright								
Wright20	0.37	0.24	0.57	6.38E-06	0.56	0.33	0.97	0.037
Train GSE10846 R-CHOP								
Wright20	0.42	0.27	0.64	4.98E-05	0.86	0.51	1.46	0.573
Wright10	0.49	0.32	0.75	1.04E-03	0.87	0.52	1.46	0.594
Wright5	0.47	0.31	0.74	9.15E-04	0.87	0.51	1.47	0.598
Dave185	0.49	0.33	0.75	7.96E-04	0.78	0.46	1.34	0.367
Dave100	0.46	0.30	0.70	3.69E-04	0.97	0.56	1.68	0.919
Dave50	0.49	0.32	0.73	5.67E-04	0.92	0.53	1.58	0.761
Dave20	0.50	0.33	0.76	1.09E-03	1.17	0.68	1.99	0.572
Dave10	0.46	0.31	0.71	3.29E-04	0.93	0.55	1.58	0.785
All185	0.49	0.33	0.73	5.67E-04	0.92	0.54	1.55	0.748
All100	0.45	0.29	0.70	3.05E-04	0.90	0.52	1.56	0.706
All50	0.45	0.29	0.69	2.22E-04	1.03	0.59	1.78	0.918
All20	0.45	0.29	0.68	1.87E-04	0.86	0.50	1.49	0.602
All10	0.44	0.29	0.67	1.42E-04	0.88	0.52	1.48	0.619
LPS Classified								
Train Wright								
Wright20	0.42	0.27	0.65	1.19E-04	0.64	0.34	1.20	0.162
Train GSE10846 R-CHOP								
Wright20	0.43	0.28	0.66	1.00E-04	0.79	0.46	1.36	0.392
Wright10	0.40	0.26	0.62	4.37E-05	0.94	0.53	1.67	0.826
Wright5	0.45	0.28	0.70	4.60E-04	0.89	0.51	1.53	0.663
Dave185	0.52	0.34	0.80	2.59E-03	0.81	0.46	1.43	0.475
Dave100	0.51	0.33	0.77	1.47E-03	0.81	0.46	1.42	0.457
Dave50	0.51	0.33	0.78	1.74E-03	0.84	0.48	1.45	0.526
Dave20	0.45	0.29	0.70	3.90E-04	1.05	0.59	1.86	0.869
Dave10	0.46	0.30	0.71	5.08E-04	1.00	0.57	1.77	0.994
All185	0.44	0.28	0.67	1.29E-04	0.83	0.49	1.42	0.496
All100	0.47	0.31	0.71	3.44E-04	0.87	0.51	1.50	0.622
All50	0.46	0.31	0.70	3.01E-04	0.90	0.52	1.53	0.687
All20	0.45	0.29	0.68	2.12E-04	0.85	0.50	1.45	0.544
All10	0.49	0.33	0.74	7.50E-04	0.78	0.44	1.36	0.375

The results obtained with classifiers trained on the Wright et al. data using 20 classifier genes were compared against those obtained with classifiers trained on the GSE10846 R-CHOP component using either the same 20 classifier genes, or a range of different classifier gene selections. Shown are the results for classifying the GSE10846 CHOP data (left) and GSE32918 (right). In each table the survival separation observed with the published GEO classes (top) was compared to the meta-classifier (middle) and the LPS (bottom). The Classifier identity, Hazard Ratio (GCB vs ABC as baseline), 95% confidence interval of the Hazard Ratio, and the resulting p-value for survival separation are shown. In the meta-classifier and LPS portions of the tables the results are shown for training on the Wright et al. data set (20 classifier genes) followed by the results for classifiers trained on the GSE10846 R-CHOP data set with different sets of classifier genes (Wright20-Wright5, Dave185-Dave10, All185-All10).

For this analysis we used the GSE10846 data set, which was generated on the Affymetrix Human Genome U133 Plus 2.0 and contained a more comprehensive representation of genes than the Wright et al. data generated on the Lymphochip platform. We trained on the R-CHOP treated component and tested on the CHOP treated component. Increasing the number of classifier genes did not improve outcome separation for either LMT_J48_RF100_SMO or LPS classifiers (Table 3). We also tested the resulting classifiers on GSE32918. This data set was best classified when using 20 classifier genes and the Wright et al. data set for training, classifiers trained on the R-CHOP component of GSE108240 data performed poorly regardless of classifier gene number (Table 3).

### Testing on Additional Data Sets

We next tested the performance of the LMT_J48_RF100_SMO meta-classifier on two additional data sets, GSE4475 and Monti et al., for which outcome data was available [10], . First in order to further evaluate the impact of training data set and classifier gene number we used the GSE4475 data set. The array platform on which GSE4475 was generated lacked probes for some classifier genes with only 16 of the 20 Wright classifier genes represented, thus gene numbers used differed from those available in GSE10846 or GSE32918. Nonetheless in this data set increasing classifier gene number again had no beneficial impact on survival separation for either LMT_J48_RF100_SMO or LPS classifiers (Table 4A). Training data set had limited impact on the performance of the classifier in evaluating GSE4475, giving p-values of 0.004 or 0.003 whether trained on Wright or GSE10846 R-CHOP data, both outperforming the significance of survival separation gained using the published classes for this data set (p-value 0.0108).

**Table 4:** Effect of data set and classifier gene number on survival.

A				
	GSE4475			
Classifier	Hazard Ratio	95% Conf Intervals		*P*-Val
GEO Published Classes	0.3622	0.1660	0.7907	0.011
LMT_J48_RF100_SMO Classified				
Train Wright				
Wright16	0.38	0.20	0.73	0.004
Train GSE10846 R-CHOP				
Wright16	0.35	0.17	0.70	0.003
Dave185	0.39	0.20	0.75	0.005
Dave100	0.42	0.22	0.81	0.010
Dave50	0.44	0.23	0.83	0.011
Dave20	0.46	0.24	0.87	0.017
Dave10	0.37	0.19	0.73	0.004
All185 (137 Actual)	0.45	0.23	0.89	0.022
All100 (69 Actual)	0.43	0.22	0.87	0.019
All50 (35 Actual)	0.37	0.18	0.76	0.007
All20 (15 Actual)	0.45	0.23	0.90	0.024
All10 (7 Actual)	0.38	0.20	0.73	0.004
LPS Classified				
Train Wright				
Wright16	0.36	0.17	0.76	0.007
Train GSE10846 R-CHOP				
Wright16	0.35	0.17	0.73	0.005
Dave185	0.46	0.23	0.93	0.031
Dave100	0.46	0.23	0.93	0.031
Dave50	0.46	0.22	0.93	0.030
Dave20	0.44	0.22	0.89	0.023
Dave10	0.35	0.17	0.72	0.004
All185 (137 Actual)	0.44	0.21	0.89	0.023
All100 (69 Actual)	0.43	0.22	0.85	0.016
All50 (35 Actual)	0.36	0.18	0.73	0.005
All20 (15 Actual)	0.46	0.24	0.89	0.021
All10 (7 Actual)	0.31	0.15	0.62	0.001
**B**				
	**Monti et al data**			
**Classifier**	**Hazard Ratio**	**95% Conf Intervals**		***P*** **-Val**
Monti Class	0.34	0.18	0.65	1.09E-03
LMT_J48_RF100_SMO	0.47	0.26	0.86	1.51E-02
LPS 0.9 MaxAvgMerge	0.49	0.26	0.93	2.95E-02

The effect of training data set and classifier gene selection was assessed on a previously unseen data set GSE4475 (**A**). Survival separation observed with the published GEO classes (top) was compared to the meta-classifier (middle) and the LPS classifier (bottom). The Hazard Ratio (GCB vs ABC as baseline), 95% confidence interval of the Hazard Ratio, and the resulting p-value for survival separation are shown. For the meta-classifier and LPS the results for training on the Wright et al. data set (20 classifier genes) are shown, followed by the results for classifiers trained on the GSE10846 R-CHOP data set with different sets of classifier genes (Wright20-Wright5, Dave185-Dave10, All185-All10). (**B**) The classifiers were also tested on Monti et al. data requiring MaxAvgMerge data processing (see Materials and Methods) to observe significant differences in survival. Results are shown for the LMT_J48_RF100_SMO and LPS (best shown) classifiers trained on Wright et al. data and using 20 classifier genes.

In contrast to GSE4475, the Monti et al. data set proved difficult to separate into classes with significant differences in survival when using probe selection and normalization parameters effective on other data sets. However using the same parameters for probe selection and normalization as reported by Monti et al. [10], regenerated significant survival separation using the LMT_J48_RF100_SMO meta-classifier, although in this instance this remained inferior to the published classes (Table 4B). The reason for this data set specific normalization and probe selection issue is not apparent, but following the procedures of Monti et al. [10], allows effective use of the data. Furthermore downstream analysis of differential gene expression further verified appropriate segregation of cases in this data set.

We conclude that a cell of origin classifier using balanced voting of LMT_J48_RF100_SMO machine-learning tools, using 20 classifier genes is applicable to data sets generated on multiple platform types, separating DLBCL into ABC and GCB classes with significant survival differences. Training on the Wright et al. data set provides better performance on the FFPE data generated on the Illumina platform, and performs well on data sets derived from fresh material generated on Affymetrix platforms. We therefore selected the combination of balanced voting of LMT_J48_RF100_SMO machine-learning tools, 20 classifier genes and training on the Wright et al. data for downstream analysis.

### Consistent Classification and Classification Confidence

**Figure 2:**
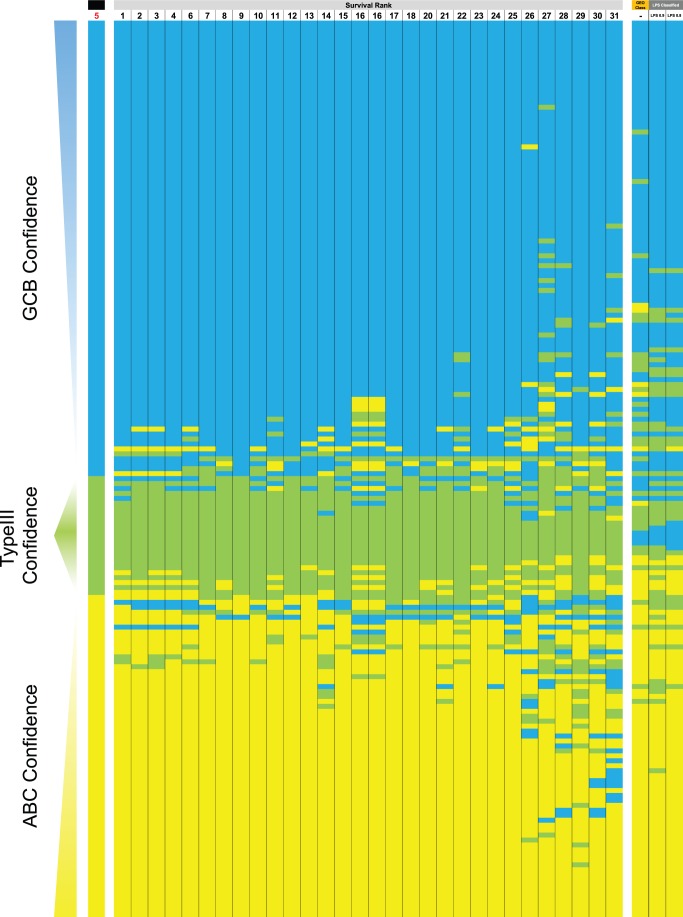
Consistent classification and classification confidence. The classes assigned by 31 tested classifiers for the GSE10846 CHOP data set are shown along with published classes in GEO and those assigned by the LPS classifier (GCB = blue, Type-III = green, and ABC = yellow). Samples are vertically ordered by class assigned by the meta-classifier LMT_J48_RF100_SMO (later referred to as “DAC”); this meta-classifier assigns confidence scores for each class, and the class with highest confidence is selected for each sample. Within each class samples are ranked by classification confidence. At either extreme, samples are ordered from high to low confidence GCB, and from low to high confidence ABC. In the Type-III category high confidence cases are shown centrally, flanked by lower confidence Type-III cases. On either side the latter are ordered by GCB or ABC signal (identified by GCB or ABC being the second highest classification confidence). The first column (labelled with black bar and red 5) identifies the classes assigned by LMT_J48_RF100_SMO, followed by results obtained for 30 other machine-learning classifiers, with the classes assigned for each case in the appropriate color. Classifiers are ranked (number above each column) from left to right according to the significance of survival separation between assigned ABC and GCB classes; note that LMT_J48_RF100_SMO was selected as the reference based on overall performance across multiple data sets, and in this data set is ranked 5^th^ (shown in red) for survival separation. On the far right the published class assignments linked in GEO to the data set (GEO class, orange bar) and classes assigned by the LPS classifier using either a 0.8 or 0.9 p-value threshold classes are shown (dark gray bars respectively).

The comparison of survival separation for ABC and GCB classes by different classifiers did not assess the effects of classifier choice on a case-by-case basis. To assess this we examined the classification choice for each case, for every classifier tested in all 3 data sets used for selecting classifiers. The resulting maps of classification choice illustrate an important point (Figure 2, Figure S1 and Table S5). A significant proportion of samples in each data set is uniformly classified by the majority of classifier implementations and thus possesses a consistent class. In contrast other samples are particularly labile and show divergent classification choices with different algorithms, classifier gene numbers or training data sets. These labile samples contribute to the differences in survival separation observed for different classifier algorithms. These results illustrate that in all data sets a “molecular gray zone” exists, and the classification of cases falling into this gray zone is susceptible to classifier choice.

The ABC and GCB classes assigned by balanced voting between LMT_J48_RF100_SMO were characterized by more significant survival separation than published classification choices for most data sets. Assessing individual case-by-case class assignments, this was not attributable to an increase in the percentage of Type-III class. Across both components of the GSE10846 data set there was little difference in the number of Type-III cases assigned: R-CHOP - 15% LMT_J48_RF100_SMO vs 14% GEO-published, CHOP –15% LMT_J48_RF100_SMO vs 17% GEO-published. The improvement in outcome separation was thus due to the selection of cases for inclusion in the ABC or GCB groups. In regard to this, LMT_J48_RF100_SMO assigned more cases to the GCB category (52% vs 46% and 51% vs 42% for R-CHOP and CHOP subsets) and fewer cases to the ABC category (33% vs 40% and 34% vs 41% for R-CHOP and CHOP subsets). Since the assignments made by LMT_J48_RF100_SMO were associated with more significant separation in survival between ABC and GCB classes, and this is the primary clinical characteristic of the cell of origin classification [1], we reasoned that this reflected improved performance and more appropriate class-assignment.

**Figure 3:**
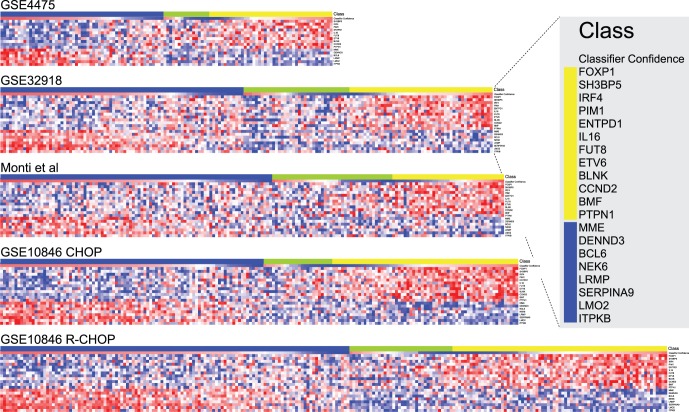
Heat-maps of classifier gene expression for data-sets used in classifier generation and testing. The data set is indicated on the left above the relevant heat-map. The LMT_J48_RF100_SMO (later referred to as “DAC”) assigned class is shown by the “Class” bar at the top of each heat-map; blue = GCB, green = Type-III and yellow = ABC. The classification confidence is shown in the “Classifier Confidence” bar under the “Class” bar (red to blue = high to low-confidence). The classifier genes are ordered vertically for each heat-map as shown in the expanded box, and the class-association of the genes is indicated by the vertical colored bar (yellow = ABC and blue = GCB). The expression values for each sample are shown as Z-scores using a blue to red color scale for low to high expression (−2 to +2).

The concept of a “molecular gray zone” was inherent in the original formulation of the cell of origin classifier where cases that fall below a defined confidence threshold for either ABC or GCB class were assigned to the Type-III/unclassified category [1], [5]. A feature of balanced voting between classifier algorithms was that a wide range of classification confidences for ABC, GCB and Type-III classes was assigned. The relationship of classifier gene expression to classifier confidence is illustrated in heat-maps of classifier genes ranked by confidence score (Figure 3). These heat-maps illustrate the weak expression of both ABC and GCB classifier genes in the Type-III/unclassified cases, and the range of classifier gene expression patterns associated with increasing classification confidence.

The balanced voting classifier provides a confidence for assignment to ABC, GCB or Type-III/unclassified and cases are assigned to the class with highest confidence, without a hard threshold. To assess whether classifier confidence assigned by the balanced voting classifier had clinical significance we examined the outcome of ABC and GCB cases by classifier confidence across all 4 data sets using hard confidence thresholds in 0.1 steps incrementing from 0.5. The results demonstrated that classifier confidence was generally associated with increasing differences in survival separation, although not in a linear fashion, since in some instances the Hazard Ratio of GCB vs ABC class was lowest for a 0.7 rather than 0.8 threshold. This was potentially attributable to the small number of highest confidence cases in any individual data set. Overall, high confidence ABC-DLBCL had a poor outcome (range of Hazard Ratios for 5 data sets 0.23 to 0.36 Table S6). However using a hard confidence threshold above 0.5 for ABC or GCB class-assignment generated an increase in Type-III/unclassified in all data sets. Since the use of the highest confidence score, without hard threshold, for class-assignment outperformed published classifications assignments in most data sets, while assigning equivalent numbers to the Type-III/unclassified category, this approach was maintained.

### A Downloadable Classifier Implementation

Routine implementation of the cell of origin classification in a clinical setting requires individual cases to be assigned to ABC, GCB or Type-III/unclassified class as they occur, rather than classification of a large collection of cases as embodied in the data sets used in this study. We therefore developed a downloadable application, featuring a simple graphical user interface, which employs the LMT_J48_RF100_SMO balanced voting approach to classify either a large collection (as in this study) or individual samples, given a background data-set from the same platform. We refer to this application as the “Diffuse Large B-cell Lymphoma Automatic Classifier” (DAC), and use this designation for the remainder of this manuscript. This application offers other groups the opportunity to directly compare their own classifications to those generated by DAC in this study. The classifier, user guide, and example data can be downloaded from: http://www.bioinformatics.leeds.ac.uk/~bgy7mc/DAC/.

### Gene Segregation Confirms Robust Classification

To verify that DAC represented a fully transferrable implementation we evaluated additional DLBCL data sets imported from GEO (Table S1). Retaining GSE10846 sub-divided by CHOP/R-CHOP treatment to account for batch effect, this increased the number of independently assessed data sets to 11. Together these represented 2030 DLBCL cases [5], [14], [21], [24], [40]. Directly linked outcome information was not available for the additional data sets in GEO, and ultimately the primary goal of the cell of origin classifier is to co-segregate cases with similar overall patterns of gene expression. We therefore used the consistency with which genes were differentially expressed between ABC and GCB class to further assess the performance of DAC.

**Figure 4:**
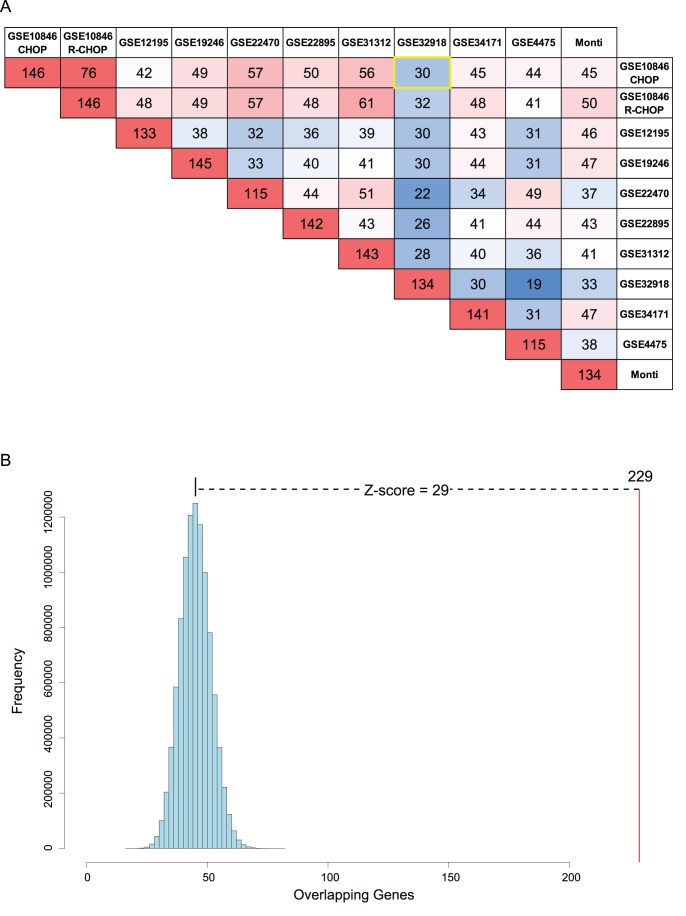
Pair-wise comparison of differentially expressed genes between all classified data-sets. (**A**) Differentially expressed genes within each class (ABC and GCB) were sequentially compared for each data set against the equivalent sets of differentially expressed genes for each other data set (ABC vs ABC and GCB vs GCB). For every comparison distributions derived from 10-million random samplings of gene sets of equivalent size were established. Comparing observed degrees of overlap against these random distributions generated Z-scores. Because the sets of differentially expressed genes for each class are of different size, the assessment generates 2 Z-scores for each class for every pair of data-sets. The Z-scores shown are the averages between the 4 Z-scores generated for each pairwise comparison (ABC and GCB in both directions of comparison). (**B**) This shows an example of the expected and observed degree of overlap between two data sets. The example shown is the degree of overlap of the ABC genes of GSE10846 CHOP (1933 genes) against the ABC genes in GSE32918 (503 genes) (yellow box in **A**). The observed degree of overlap (229 genes, 45% of differentially expressed ABC genes in GSE32918) is 29 standard deviations away from the expected random degree of overlap.

We identified differentially expressed genes between ABC and GCB class defined by DAC for each data set. We refer to these differentially expressed genes, as “class-associated”. We performed a pairwise comparison of the resulting lists of class-associated genes for all data set combinations, using a hypergeometric test (Figure 4A). For each pairwise comparison 10^7^ random gene lists of equivalent size were used to establish the expected distribution against which the observed overlaps were assessed. This analysis showed that differentially expressed genes associated with ABC and GCB class defined by DAC were very similar for each data set. Two FFPE derived data sets, GSE32918 and GSE31312, are included in this analysis [22], [23]. GSE31312, derived from an Affymetrix platform, showed amongst the highest concordances with GSE10846 generated from fresh frozen material on the same platform. While the data derived from FFPE material on an Illumina platform (HumanRef-8 V3) [22], showed lower concordance, the overall similarity is still highly significant (Figure 4B). The observed variation in overlap is not surprising given both the difference in case series and platform type. Thus DAC generates comparable segregation of gene expression for ABC and GCB DLBCL in data sets derived from different platform types, case series, and operators.

### Establishing DLBCL Meta-profiles

We next proceeded to a more refined comparative analysis of gene expression, assessing consistent associations of individual genes with the primary cell of origin classes, ABC and GCB DLBCL. We compared the lists of class-associated genes for all 11 data sets, maintaining the sub-division of GSE10846 by CHOP/R-CHOP treatment, ranking genes first by the number of data sets in which the genes were class-associated and second by the median normalized fold change (Table 5 and Table S7). A threshold of 6 or more data sets was chosen to define lists of genes that are differentially expressed and class-associated in at least half the data sets. This provides a critical additional variable, the consistency of class-association, which differentiates these lists from prior assessments made on individual data sets. We refer to these lists as “meta-profiles”, which are composed of 414 genes for ABC-DLBCL and 415 for GCB-DLBCL.

**Table 5:** ABC and GCB DLBCL meta-profiles.

Up-regulated in ABC				Up-regulated in GCB			
Gene	Classifier Gene	Median Normalised FC	Number Of Files	Gene	Classifier Gene	Median Normalised FC	Number Of Files
ZBTB32		0.69	11	MME	Yes	0.67	11
KCNA3		0.59	11	LMO2	Yes	0.66	11
CYB5R2		0.58	11	SPINK2		0.62	11
CCND2	Yes	0.56	11	STAG3		0.56	11
IRF4	Yes	0.54	11	LRMP	Yes	0.41	11
PHF16		0.52	11	ASB13		0.41	11
FAM46C		0.52	11	AUTS2		0.40	11
BATF		0.52	11	MAPK10		0.40	11
PIM2		0.46	11	BCL6	Yes	0.40	11
TNFRSF13B		0.44	11	SLC12A8		0.37	11
FUT8	Yes	0.41	11	PLEKHF2		0.36	11
SH3BP5	Yes	0.40	11	SSBP2		0.35	11
ADTRP		0.39	11	DENND3	Yes	0.31	11
ENTPD1	Yes	0.37	11	FADS3		0.30	11
TCF4		0.34	11	ITPKB	Yes	0.28	11
ARID3A		0.33	11	PTK2		0.24	11
HSP90B1		0.31	11	HIP1R		0.23	11
PIM1	Yes	0.31	11	STS		0.20	11
BCL2L10		0.30	11	VGLL4		0.16	11
BLNK	Yes	0.30	11	SULT1A1		0.15	11
CREB3L2		0.28	11	MYBL1		0.65	10
MAN1A1		0.26	11	TTC9		0.34	10
CFLAR		0.20	11	ZPBP2		0.46	9
CLINT1		0.13	11	FNDC1		0.43	9
BSPRY		0.45	10	SNX22		0.32	9
ARID3B		0.28	10	EEPD1		0.29	9
ATP13A3		0.19	10	ANKRD13A		0.24	9
C1ORF186		0.54	9	SERPINA9	Yes	0.93	8
TOX2		0.53	9	LINC00487		0.72	8
CLECL1		0.49	9	LOC285286		0.43	7
LRRC33		0.42	9	SNX29P1#SNX29P2		0.87	6
FOXP1	Yes	0.38	9	LOC440864		0.55	6
ZNF385C		0.34	9	C12ORF77		0.36	6
CCDC50		0.24	9				
PARP15		0.53	8				
MPEG1		0.43	8				
TBC1D27	Yes	0.42	8				
FAM108C1		0.41	8				
ISY1#RAB43		0.17	6				

Genes shown are differentially expressed and up-regulated in the indicated class in all data sets (shown for > = 6) that have a corresponding probe (ABC (**left)** and GCB (**right**)). **ClassifierGene**: genes used in classifier; **Median NFC**: median normalised fold change (0–1 for differentially expressed genes); **NumFiles**: number of files in which gene is differentially expressed and upregulated in the indicated class. See Table S7 for complete lists.

Excluded Wright classifier genes (Tables S2 and S7) provided an initial means for assessment of these meta-profiles [2]. Of seven Wright classifier genes that were not represented on the Illumina HumanRef-8 WG-DASL v3.0 array, three had RefSeq IDs, and were included on other platforms. *MYBL1*, a GCB classifier gene, was represented in 10 data sets, in all 10 data sets it was strongly GCB class-associated, and ranked 21^st^ in the meta-profile. *TBC1D27*, an ABC classifier gene, was represented in 8 data sets; in all of these it was strongly ABC class-associated and ranked 103^rd^ in the ABC meta-profile. The third excluded classifier gene *IGHM* was also represented on 10 data sets, and while class-associated in 7 of these data sets and hence ranked 172^nd^, it was the single most strongly ABC-DLBCL associated gene when considering median normalized fold-change alone. Thus the appropriate class-segregation of these excluded Wright classifier genes, also confirms the internal consistency of classification with DAC.

### Meta-profiles Identify Highly Class-associated Transcription Factors

The meta-profiles are informed by consistency of expression across multiple different data sets. This provides an important variable for assessing the likely significance of a gene in the lymphoma class. Notably of the 414 genes in the ABC-DLBCL meta-profile only 24 genes were class-associated in 11/11 data sets. Microarray platforms differ in the selection of probes and hence the ability to assess individual genes. When taking differential representation of genes into account, 42 genes were ABC class-associated in all data sets in which probes for the gene were present on the platform used (100% class-association). Equally for GCB-DLBCL 20 genes were class-associated in 11/11 data sets, with 35 being 100% class-associated when accounting for gene representation on platforms used.

A notable feature was that the top three genes (*ZBTB32*, *KCNA3* and *CYB5R2*) in the ABC-DLBCL meta-profile were not primary classifier genes. In contrast for GCB-DLBCL the classifier genes *MME* (CD10) and *LMO2* topped the ranking. It was also notable that the most consistent ABC-DLBCL meta-profile genes included the transcriptional regulators *BATF*, *TCF4*, *ARID3A*, and *CREB3L2* in addition to *ZBTB32* and the primary classifier genes *IRF4* and *FOXP*1.

### Enrichment of Appropriate Gene Signatures for Class

We reasoned that if the data sets were appropriately classified then meta-profiles should be enriched for genes that overlap in a statistically significant fashion with gene signatures representing lists of genes defined in previous work linked to cell of origin class or molecular pathogenesis (i.e. contain more signature genes than expected by chance for a list of equivalent number). Furthermore if this condition were met, and since the meta-profiles were uniquely informed by consistency of gene expression across multiple data sets, then analysis of gene signature databases would additionally form the basis for identifying novel associations relevant to disease biology. The databases MSigDB, GeneSigDB, as well as the SignatureDB of the Lymphoma Leukemia Molecular Profiling Project, provide an extensive compendium of gene signatures/sets related to particular cell states, pathways, or gene features [27], [28], [41]. We used these compendia, and a hypergeometric test, to provide an unbiased assessment of the enrichment of gene sets/signature in the meta-profiles. For these analyses all classifier genes were excluded, since these inevitably show bias in association, and the use of only 20 classifier genes provides a substantial advantage in this respect. Amongst all 12323 signatures tested 903 showed significant enrichment, after correction for multiple testing in the ABC meta-profile and 1513 in the GCB meta-profile (Table 6 and 7, and Table S8). In each case (ranking by FDR corrected p-value) extended signatures of the ABC- and GCB-DLBCL subtypes were the most enriched of all signatures tested: ABCgtGCB_U133AB (FDR corrected p-value 5.61E-235) and GCB_gt_ABC_U133plus (FDR corrected p-value 4.59E-214) [41]. These signatures showed a 61.85% and 54.55% overlap of genes with ABC meta-profile and GCB meta-profile respectively. Other cell of origin related signatures showed similar high but not complete degrees of overlap, such as ABC_gt_GCB_PMBL_MCL_BL_U133AB (80.43% overlap and FDR corrected p-value 2.68E-55) for the ABC meta-profile, and GC_B_cell_U133Plus (25.31% overlap and FDR corrected p-value 3.05E-70) for the GCB meta-profile.

**Table 6:** Selected gene signatures significantly enriched or depleted in the ABC meta-profile.

Enriched										
Gene Signature	Overlapping	GeneSigSize	randomAvg	randomSD	%Overlap	Zscore		FDR		Source
ABCgtGCB_U133AB	167	270	4.52	2.10	61.85	77.45		5.61E-235 **		SignatureDB
ABC_gt_GCB_PMBL_MCL_BL_U133AB	37	46	0.77	0.87	80.43	41.65		2.68E-55 **		SignatureDB
NFkB_Up_all_OCILy3_Ly10	13	64	1.07	1.02	20.31	11.65		9.07E-09 **		SignatureDB
MYD88_Ngo_etal	21	266	4.45	2.08	7.89	7.96		6.83E-07 **		SignatureDB
NFkB_Up_HBL1	18	211	3.53	1.85	8.53	7.80		2.28E-06 **		SignatureDB
V$NFKB_Q6_01	18	231	3.86	1.94	7.79	7.29		8.17E-06 **		MSigDB_C3
NFkB_Up_bothOCILy3andLy10	8	37	0.62	0.78	21.62	9.48		1.33E-05 **		SignatureDB
chr3q29	7	54	0.91	0.94	12.96	6.47		1.22E-03 **		MSigDB_C1
chr18p11	8	75	1.25	1.11	10.67	6.09		1.38E-03 **		MSigDB_C1
chr3q13	7	86	1.44	1.19	8.14	4.69		0.01 *		MSigDB_C1
chr3q21	7	99	1.66	1.27	7.07	4.19		0.02 *		MSigDB_C1
chr3p21	11	237	3.96	1.96	4.64	3.58		0.03 *		MSigDB_C1
chr18q21	6	82	1.37	1.16	7.32	3.99		0.04 *		MSigDB_C1
KEGG_FOCAL_ADHESION	7	198	3.31	1.80	3.54	2.06		0.27		MSigDB_C2
TGGAAA_V$NFAT_Q4_01	40	1883	31.48	5.35	2.12	1.59		0.33		MSigDB_C3
chr12p13	6	201	3.36	1.81	2.99	1.46		0.45		MSigDB_C1
CROONQUIST_STROMAL_STIMULATION_UP	2	60	1.00	0.99	3.33	1.00		0.64		MSigDB_C2
SUNG_METASTASIS_STROMA_UP	3	110	1.84	1.34	2.73	0.87		0.66		MSigDB_C2
KEGG_ECM_RECEPTOR_INTERACTION	2	84	1.40	1.17	2.38	0.51		0.75		MSigDB_C2
**Depleted**										
**Gene Signature**	**Overlapping**	**GeneSetSize**	**randomAvg**	**randomSD**	**%Overlap**		**Zscore**		**FDR**	**GeneSet**
GCB_gt_ABC_U133plus	0	297	4.97	2.20	0.00		−2.26		0.07	SignatureDB
Stromal-1_DLBCL_survival_predictor	0	260	4.35	2.06	0.00		−2.12		0.12	SignatureDB
GC_B_cell_U133Plus	3	324	5.42	2.29	0.93		−1.06		0.59	SignatureDB
chr17q24	0	42	0.70	0.83	0.00		−0.85		0.80	MSigDB_C1
chr2q23	0	22	0.37	0.60	0.00		−0.61		0.86	MSigDB_C1

Gene signature enrichments in the meta-profiles were assessed with a hypergeometric test. Shown are selected signatures discussed in the text, including those related to the reciprocal class, a comprehensive list is provided in Table S8. ***Gene Signature***: name of signature, ***Overlapping*** *:* how many of the ABC meta-profile genes overlap with signature, ***GeneSigSize***: number of genes in gene signature (after classifier genes are removed), ***randAvg/randSD***: average/standard-deviation overlap from distribution containing 1 million random samplings, ***%Overlap***: percentage of gene signature that overlaps with meta-profile, ***Zscore***: standard score of observed normalised against random distribution, ***FDR***: Benjamini-Hochberg false discovery rate, ***Source***: gene signature origin. Shown is a selection of gene signatures (see Table S8 for complete list). ** FDR <0.01, * FDR <0.0.

**Table 7:** Selected gene signatures significantly enriched or depleted in the GCB meta-profile.

Enriched										
Gene Signature	Overlapping	GeneSigSize	randomAvg	randomSD	%Overlap	Zscore		FDR		Source
GCB_gt_ABC_U133plus	162	297	4.98	2.20	54.55	71.46		4.59E-214 **		SignatureDB
GC_B_cell_U133Plus	82	324	5.43	2.30	25.31	33.33		3.05E-70 **		SignatureDB
Stromal-1_DLBCL_survival_predictor	61	260	4.36	2.06	23.46	27.52		3.92E-49 **		SignatureDB
TGGAAA_V$NFAT_Q4_01	68	1883	31.57	5.36	3.61	6.80		1.15E-07 **		MSigDB_C3
KEGG_FOCAL_ADHESION	18	198	3.32	1.80	9.09	8.16		4.99E-07 **		MSigDB_C2
CROONQUIST_STROMAL_STIMULATION_UP	10	60	1.01	0.99	16.67	9.05		3.19E-06 **		MSigDB_C2
KEGG_ECM_RECEPTOR_INTERACTION	11	84	1.41	1.18	13.10	8.16		7.88E-06 **		MSigDB_C2
SUNG_METASTASIS_STROMA_UP	12	110	1.84	1.34	10.91	7.57		1.51E-05 **		MSigDB_C2
chr12p	2	6	0.10	0.31	33.33	6.03		0.04 *		MSigDB_C1
chr17q24	4	42	0.70	0.83	9.52	3.96		0.04 *		MSigDB_C1
chr2q23	3	22	0.37	0.60	13.64	4.37		0.05 *		MSigDB_C1
MYD88_Ngo_etal	10	266	4.46	2.08	3.76	2.66		0.09		SignatureDB
chr3q13	4	86	1.44	1.19	4.65	2.15		0.24		MSigDB_C1
chr3q21	4	99	1.66	1.27	4.04	1.84		0.31		MSigDB_C1
V$NFKB_Q6_01	7	231	3.87	1.94	3.03	1.61		0.34		MSigDB_C3
NFkB_Up_HBL1	5	211	3.54	1.86	2.37	0.79		0.59		SignatureDB
chr3q29	1	54	0.91	0.94	1.85	0.10		0.81		MSigDB_C1
NFkB_Up_all_OCILy3_Ly10	1	64	1.08	1.03	1.56	−0.07		0.84		SignatureDB
**Depleted**										
**Gene Signature**	**Overlapping**	**GeneSetSize**	**randomAvg**	**randomSD**	**%Overlap**		**Zscore**		**FDR**	**GeneSet**
ABCgtGCB_U133AB	0	270	4.53	2.10	0.00		−2.16		0.07	SignatureDB
chr3p21	2	237	3.98	1.97	0.84		−1.00		0.55	MSigDB_C1
chr18p11	0	75	1.26	1.11	0.00		−1.13		0.59	MSigDB_C1
ABC_gt_GCB_PMBL_MCL_BL_U133AB	0	46	0.77	0.87	0.00		−0.89		0.73	SignatureDB
NFkB_Up_bothOCILy3 andLy10	0	37	0.62	0.78	0.00		−0.79		0.78	SignatureDB
chr18q21	1	82	1.38	1.16	1.22		−0.32		0.81	MSigDB_C1

Gene signature enrichments in the meta-profiles were assessed with a hypergeometric test. Shown are selected signatures discussed in the text, including those related to the reciprocal class, a comprehensive list is provided in Table S8. ***Gene Signature***: name of signature, ***Overlapping*** *:* how many of the GCB meta-profile genes overlap with signature, ***GeneSigSize***: number of genes in gene signature (after classifier genes are removed), ***randAvg/randSD***: average/standard-deviation overlap from distribution containing 1 million random samplings, ***%Overlap***: percentage of gene signature that overlaps with meta-profile, ***Zscore***: standard score of observed normalised against random distribution, ***FDR***: Benjamini-Hochberg false discovery rate, ***Source***: gene signature origin. Shown is a selection of gene signatures (see Table S8 for complete list). ** FDR <0.01, * FDR <0.05.

We also considered signature enrichment for differentially expressed genes for each class in each data set independently. This approach assesses the consistency of signature enrichment across data sets (Table S9), and emphasizes the co-ordinated expression of components of a signature, rather than the expression of individual genes. In this analysis extended ABC- and GCB-DLBCL signatures again emerged as most highly and consistently enriched in all data sets: ABC_gt_GCB_U133AB (11/11 data sets, average p-value 9.42E-67) for ABC class, and GCB_gt_ABC_U133plus (11/11 data sets, average p-value 3.10E-78) for GCB class. Furthermore these analyses confirm that the ABC and GCB class-associated genes observed in FFPE data show similar patterns of molecular signature enrichments to those observed in data sets derived from fresh material (See GSE32918 and GSE31312 columns in Table S9). We can conclude that DAC allows robust class-specific segregation of gene expression regardless of data source and platform type.

### Oncogenic Pathway Enrichment in ABC-DLBCL

Several oncogenic pathways have been established for ABC-DLBCL [6]. Focusing on gene signatures that were selectively enriched in the ABC but not the GCB meta-profile, amongst the most enriched signatures were those related to NFkB or MYD88 activation: NFkB_Up_all_OCILy3_Ly10 ranked 37^rd^ (ranking by FDR adjusted p-value), MYD88_Ngo_etal ranked 72^nd^, NFkB_Up_HBL1 ranked 85^th^, and NFkB_Up_bothOCILy3andLy10 ranked 113^th^ [35], [42], [43], [44], [45].

Other signatures of NFkB activity have been defined in distinct cellular contexts. Our analysis allowed a ranking of the relative enrichment of these signatures across both the meta-profiles and individual data sets. Of 46 signatures related to NFkB, 8 were enriched in the ABC but not GCB meta-profiles and of these 3 (“NFkB_Up_all_OCILY3_LY10”, “NFkB_Up_HBL1”, “NFkB_Up_bothOCILY3andLY10”) were enriched in 11/11 individual data sets (Table S8 and S9) [36]. In addition to the 3 classifier genes that are NFkB targets (*CCND2*, *IRF4* and *PIM1*) the ABC meta-profile genes contributing to the enrichment of these three NFkB signatures were: *ADAM8, BATF, CCL22, CCR7, CD44, CFLAR, ELL2, EPHB1, GPR183, HCK, IL10, IL12A, LITAF, LYN, MARCKS, MIR155HG, NOSIP, PLAGL1, PLK3, RAB7L1, RNF183, SLC38A5, SMARCA2, SNX18, STAT3, TCEB3*. Since these signatures were generated from ABC-DLBCL cell lines there is potential circularity in detecting enrichment in the ABC subset. However independent evidence for the specific role of NFkB target genes in the ABC-DLBCL meta-profile was also provided by the enrichment of gene sets linked to promoters with evolutionarily conserved transcription factor motifs contained in the C3 component of MSigDB [29]. The most enriched such gene set in the ABC-DLBCL profile was for NFkB binding sites, V$NFKB_Q6_01 (7.79% overlap, FDR corrected p-value = 8.17E-6). Of the 19 genes (excluding classifier gene *BMF*) contributing to this enrichment only two overlapped with those linked to the experimental NFkB target lists (*BATF* and *IL12A*). The enrichment of the NFkB motif gene set in the ABC meta-profile contrasted with the GCB meta-profile in which the most significantly enriched MSigDB_C3 gene sets were FOXO motif variants as well as the NFAT motif (TGGAAA_V$NFAT_Q4_01 3.61% overlap, FDR corrected p-value = 1.15E-07). Notably the NFkB and NFAT gene sets were not enriched in the reciprocal meta-profiles indicating enrichment specific to DLBCL class. Since NFkB and NFAT are two of the major transcription factor targets downstream of surface receptor signaling in B-cells, these results support the concept that differential usage of these pathways is a central feature separating ABC- and GCB-DLBCL.

### Association of GCB-DLBCL with Stromal and Focal Adhesion Signatures

For GCB-DLBCL a distinctive feature was association with stromal signatures. The most significant enrichment was observed for the signature Stromal-1_DLBCL_survival_predictor (ranked 4^th^, 23.5% overlap, FDR corrected p-value = 3.92E-49) (Table 7) [5]; and amongst individual data sets this signature was enriched in 10/11. The strong class association was further underlined by the fact that this signature shared no overlapping genes with the ABC-DLBCL meta-profile, representing a significant depletion (Zscore = −2.12) (Table 6). Indeed other stromal signatures such as “CROONQUIST_STROMAL_STIMULATION_UP” (16.67% overlap, FDR corrected p-value 3.19E-06) and “SUNG_METASTASIS_STROMA_UP” (10.91% overlap, FDR corrected p-value 1.51E-05) were also enriched in the GCB meta-profile. Amongst KEGG pathway signatures 9 were significantly enriched in the GCB-DLBCL meta-profile, of which the 2 most significantly enriched were: “KEGG_FOCAL_ADHESION” (9.09% overlap, FDR corrected p-value 4.99E-07), “KEGG_ECM_RECEPTOR_INTERACTION” (13.10% overlap, FDR corrected p-value 7.88E-06). Thus expression of genes associated with stromal interactions is a particular and consistent characteristic of GCB-DLBCL.

### Gene Expression Signatures of DLBCL Show Consistent Associations with Chromosomal Regions

**Figure 5:**
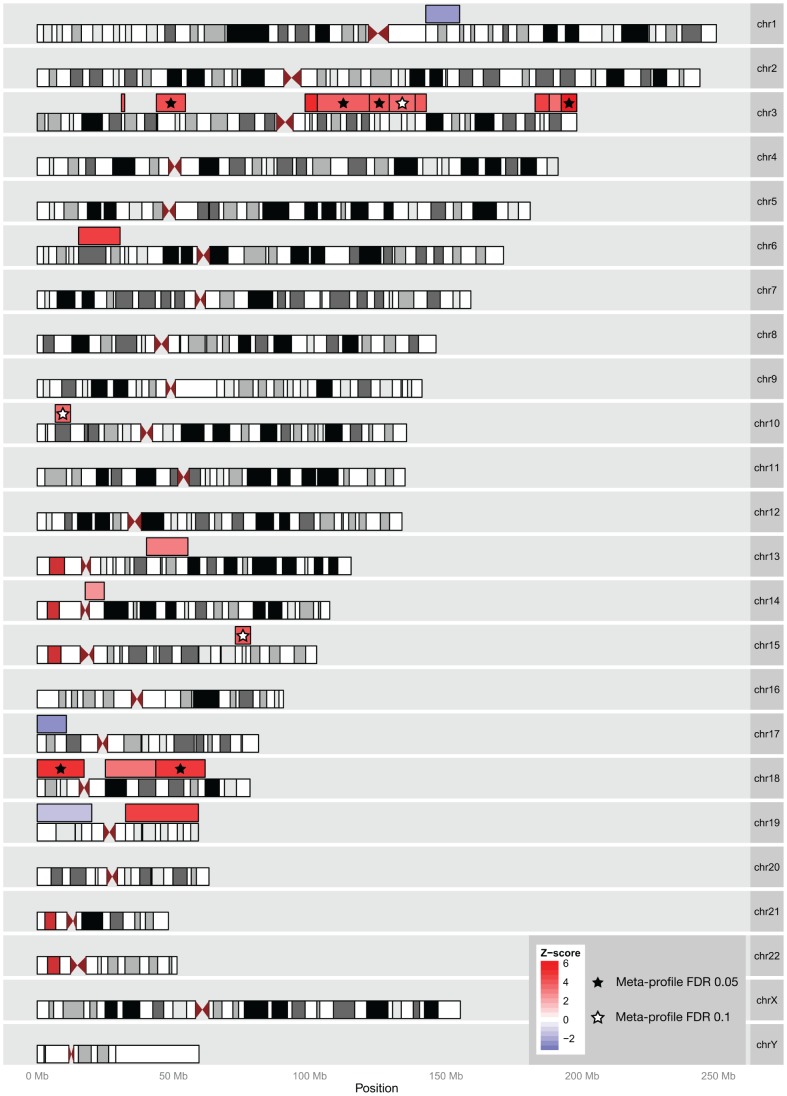
Chromosome cytoband enrichment/depletion in the ABC class. Human chromosomal cytobands are depicted using gray scales, with chromosomes displayed vertically in numerical order. Enrichment or depletion of cytobands was assessed using a hypergeometric test and the MSigDB C1 component against the meta-profiles and across differentially expressed genes for each data set individually. Observed enrichments and depletions are shown as the average Z-scores with red to blue color scale (average Z-score = +6 to −2) as indicated in the insert. These are derived from analyses of individual data sets (Table S9), with significant enrichment in >5/11 data sets used as threshold for inclusion. Regions additionally identified as significantly enriched in the ABC meta-profile (Table S8) are indicated with a star (FDR corrected p-value <0.05 = black star, FDR corrected p-value <0.1 = white star).

Recurrent cytogenetic abnormalities characterise DLBCL and show association with cell of origin class and outcome [4], [46]. Therefore a conspicuous feature of the ABC and GCB meta-profiles was differential enrichment of gene signatures associated with chromosomal regions/cytobands. Of the 326 chromosomal (chr) cytoband signatures contained in the C1 component of MSigDB, 6 were significantly enriched in the ABC meta-profile, while 3 (chr12p, chr17q24 and chr2q23) were enriched in the GCB meta-profile. Four of the 6 enriched cytobands in the ABC meta-profile are on chr3 (Figure 5 and Table S8), and 2 on chr18 (chr18p11 and chr18q21). A similar bias toward chr3 enrichment was evident when each data set was individually assessed. 22 cytobands were enriched in 5 or more data sets, 10 of these were derived from chr3 (chr3-p21, p23, q12, q13, q21, q22, q23, q27, q28 and q29) of these 3q13 and 3q29 were the most consistent. Amongst individual data sets three chr18 cytobands were enriched in 5 or more data sets, chr18p11, chr18q21 and chr18q12. These patterns largely reflect regions of recurrent chromosomal aberrations [4], [46], and provide evidence for the particular significance of chr3 and chr18 deregulation in ABC-DLBCL.

## Discussion

Despite the established nature of the cell of origin classification a comparative analysis of gene expression across multiple data sets classified with the same algorithm has not been published. To allow such an analysis we have developed a robust classifier based on 20 genes, described in the original Wright classifier implementation [2], and represented on multiple platform types. The use of a small number of classifier genes is an important feature since primary classifier genes show intrinsic bias and need to be disregarded for downstream analyses. Since the inception of the cell of origin classification, variations have been described that employ larger numbers of classifier genes [5], [9]. However, to our knowledge, there has been no direct comparison of whether large numbers of classifier genes produce consistent improvement in classification. Here we have directly addressed this question and using a range of different classifier gene choices we find no consistent benefit of using larger numbers of classifier genes. Indeed in several instances classifiers relying on greater numbers of classifier genes generate ABC- and GCB-DLBCL classes with less significant survival separation.

An issue of primary importance in the evaluation of a test is the performance against a “gold standard” and the choice of metric that is used to assess performance. The most significant clinical feature of the cell of origin classification is the ability to separate DLBCL into two major subgroups ABC and GCB with different survival [1]. In assessing the performance of classifiers we therefore used survival separation as the metric. The “gold standard” can be seen either as the LPS classifier described by Wright et al. [2], or the classes assigned by subsequent extension of this classifier to include more classifier genes [5]. While the LPS classifier effectively separates multiple data sets from fresh frozen material into classes with significant survival separation, classification choices made by machine-learning tools using 20 classifier genes generate more significant differences in survival separation than those made by LPS or the published class assignments for 4/5 data sets (considering the CHOP and R-CHOP components of GSE10846 separately), the original data set of Monti et al. being the exception [10]. Furthermore our selected classifier can distinguish prognostic groups in an FFPE derived data set generated on an Illumina platform [22], while the LPS classifier was much less effective in this data set. Another FFPE derived data set included in this analysis, GSE31312, was generated on Affymetrix HG-U133 Plus 2.0 GeneChips using FFPE derived samples [23]. This was also readily classified by DAC and at the level of segregation of gene expression was amongst the most concordant with GSE10846.

An important observation emerging from this study is the existence of an extended molecular gray zone, representing cases whose classification is sensitive to the type of classifier implementation used. A substantial group of cases in each data set was equivalently classified by most classifier implementations and thus had a consistent class. In contrast the differences in outcome separation observed were attributable to cases that had more marginal expression of classifier genes and moved been class in a fashion dependent on classifier implementation. While a “molecular gray zone” was inherent in the cell of origin classification, and in its original form encompassed the Type-III or unclassifiable cases [1], [2], when considering the choices made by different classifier implementations the extent of this molecular gray zone was greater. The concept of cases that do not fall neatly into one or other diagnostic group is familiar and indeed is encompassed in distinct categories of the WHO lymphoma classification [47]. It is therefore no surprise that gene expression profiling is similarly subject to ambiguity, and that choice of weighting for individual genes results in differential classification of some cases. Such ambiguity may be resolved in future by using more discrete variables, for example the presence or absence of particular pathway mutations. Nonetheless gene expression based classification schemes are likely to continue to provide additional important information, since they can assess the combined impact of multiple molecular abnormalities acting within a tumour cell population to drive a predominant phenotype. An analysis such as that performed in this work can provide the basis on which to select between individual classifier implementations. In making this selection a metric needed to be chosen. We opted for the difference in overall survival between the principal cell of origin classes assigned by each classifier, while ensuring that this did not come at the expense of greater inclusion into the Type-III/unclassified category. We used the consistency of class-associated gene expression as a supporting feature. As illustrated in this work it is possible to improve on classification choices using different implementations, while remaining within an existing classification paradigm. While such choices have limited impact in the research setting, if classification based on gene expression profile is in future linked to treatment choice then classifier implementation will become a significant consideration. It could be argued that a systematic evaluation of classifier implementations is advisable in such circumstances, where sufficient publically available data sets are available.

The development of meta-profiles representing the most consistent differentially expressed genes between ABC- and GCB-DLBCL is significant since these gene lists are uniquely informed by the consistency of differential gene expression between multiple data sets. Indeed limited numbers of genes were detected as ABC- or GCB-associated in all data sets, and these genes are likely to be enriched for core regulators. Amongst the transcription factors most consistently linked to the ABC-subset is *BATF*, an NFkB target gene that has recently been identified as a key partner of IRF4 [48], [49], [50], [51]. This is of particular interest since ABC-DLBCL and myeloma show non-oncogenic addiction to IRF4 function [52], [53]. IRF4 depends on partner transcription factors to occupy DNA, the canonical partners being ETS-factors SPIB or PU.1 in B-cells; in contrast BATF plays a dominant role in T-cells [48], [50], [51]. IRF4 is itself an NFkB target gene, which is a principle mechanism proposed for its expression in ABC-DLBCL [54]. *BATF* expression in ABC-DLBCL may be explained in a similar fashion since it features on multiple NFkB signature lists, including the MSigDB signature V$NFKB_Q6_01. Indeed *BATF* is induced transiently in activated B-cells during CD40 driven plasma cell differentiation [55]. Amplification of chr19q as well as translocations can deregulate *SPIB*, leading to functional addiction to this transcription factor [4], [52]. It will therefore be interesting to assess the relative contributions of SPIB and BATF to IRF4 function in ABC-DLBCL.

Another feature of the ABC-DLBCL meta-profile was the fact that three genes, *ZBTB32*, *KCNA3* and *CYB5R2* show a more consistent class-association than any of the primary classifier genes. *ZBTB32* encodes a transcription factor, also known as Repressor of GATA (ROG), with functions in T-cell activation and differentiation [56]. The role of this gene in B-cell differentiation is somewhat enigmatic, but *ZBTB32* has recently been identified as a repressor of transcription rapidly induced during murine B-cell differentiation, which can co-operate with BLIMP1 in silencing *CIITA* [57]. The latter is essential for MHC class-II expression, and shows a weak class-association with GCB-DLBCL. Expression of ZBTB32 and repression of *CIITA* may thus contribute to immune-evasion in ABC-DLBCL [54], [58]. *KCNA3* (also known as Kv1.3) encodes a potassium channel, which is expressed in effector memory T-cells and memory B-cells [59], [60]. KCNA3 has been identified as a potential target for therapy in autoimmune disease [61], and several strategies for inhibition have been developed in this context [59]. The strong and consistent expression of *KCNA3* identifies this as an intriguing candidate for a novel therapeutic approach in ABC-DLBCL.

Enrichments of gene signatures derived from previous studies [29], [35], [42], [43], [44], [45], across meta-profiles and across individual data sets provides a resource for the identification of class/signature/gene associations of relevance to lymphoma pathogenesis and further confirmed the validity of classifications. A striking feature was the ability to identify gene sets associated with known regions of chromosomal deregulation in DLBCL. Several studies have assessed the contribution of copy number alterations to deregulated gene expression [46], [62], with a notable recent paper using integrated analysis to assess the contribution of multiple regions of copy number change to deregulation of cell-cycle checkpoints [26]. Our comparative analysis has allowed a distinct approach identifying over-represented chromosomal cytobands by the consistency of gene expression across multiple data sets. This approach emphasised the particular importance of chr3 and chr18 to ABC-DLBCL, and also identified genes in chromosomal regions as potential drivers of pathogenesis. An example of this was evident in chr18q21. Amplification of chr18q is common in ABC-DLBCL and associated with aberrant expression of *BCL2* [4], [26]. Identifying differential expression of *BCL2* is complicated by the fact that *BCL2* translocations are a common feature of GCB-DLBCL, but nonetheless *BCL2* was ABC class-associated. However, the most consistently differentially expressed gene from chr18q21 in ABC-DLBCL was *TCF4*. This gene encodes a transcription factor also known as E2-2, which can drive *SPIB* expression and co-operate with SPIB to regulate the transcriptional program of plasmacytoid dendritic cells [63], [64]. Notably several dendritic cell signatures are enriched in the ABC-DLBCL meta-profile including Dendritic_cell_CD123pos_blood, CD123 being a surface marker of plasmacytoid dendritic cells, ranked 6^th^ of all signatures in the ABC meta-profile (12.38% overlap, FDR corrected p-value = 8.95E-18). Thus in addition to the deregulation of BCL2, amplification of chr18q21 is likely to contribute to the consistent expression of *TCF4*, and hence to establishing the transcriptional network of ABC-DLBCL.

In conclusion, the generation of the robust classifier algorithm, DAC, provides a tool with which to consistently classify DLBCL cases regardless of microarray platform type. It has potential applications in the research and clinical setting, since it is designed, and is currently being used, to allow real-time assessment of individual incident cases. Currently real-time classification of DLBCL cases into molecular classes does not affect primary clinical management decisions, but in future this may change. The analysis we have performed highlights the issues surrounding the effect that classifier choice can have on class assignment, and argues for a robust analysis of classifier algorithm in such settings. The development of this classifier has allowed the generation of a useful resource in which the consistency of class-associated gene expression provides a method for identifying associations of relevance to disease biology, and in particular highlights transcription factors operating in ABC-DLBCL.

## Supporting Information

Figure S1 **Consistent classification and classification confidence.** This accompanies Figure 2. The classes assigned by 31 tested classifiers for the (**A**) GSE10846 CHOP, (**B**) GSE10846 R-CHOP, (**C**) GSE32918 data set are shown along with published classes in GEO and those assigned by the LPS classifier (GCB = blue, Type-III = green, and ABC = yellow). Part **(A)** reproduces the data shown in Figure 2 and is included for completeness and to allow direct comparisons. As in Figure 2, samples are vertically ordered by the class given by the meta-classifier LMT_J48_RF100_SMO (later referred to as “DAC”); this meta-classifier assigns confidence scores for each class, and the class with highest confidence is selected for each sample. Within each class samples are ranked by classification confidence. At either extreme, samples are ordered from high to low confidence GCB, and from low to high confidence ABC. In the Type-III category high confidence cases are shown centrally flanked by lower confidence Type-III cases. On either side the latter are ordered by GCB or ABC signal (identified by GCB or ABC being the second highest classification confidence). The first column (labelled with black bar and red 5) identifies the classes assigned by LMT_J48_RF100_SMO, followed by results obtained for 30 other machine-learning classifiers, with the classes assigned for each case in the appropriate color. Classifiers are ranked (number above each column) from left to right according to the significance of survival separation between assigned ABC and GCB classes; note that LMT_J48_RF100_SMO was selected as the reference based on overall performance across multiple data sets, and in this data set is ranked 5^th^ (shown in red) for survival separation. On the far right the published class assignments linked in GEO to the data set (GEO class, orange bar) and classes assigned by the LPS classifier using either a 0.8 or 0.9 p-value threshold classes are shown (dark gray bars respectively).
(PDF)

Table S1 **Dataset details.** This table provides details of the datasets used, showing the GEO accession number where applicable, the title of the associated publication, data set size, array type (Chip Number), presence of associated survival information (Yes/No), and the stages of the evaluation for which the data set was used.
(XLSX)

Table S2 **Classifier genes.** This table lists the original Wright et al. classifier genes and their cell of origin class association. Genes used in classifier development are shown in bold, asterisks identify Lymphochip features for which an official gene symbol was not identified. The *GeneSelections* work sheet shows the extended classifier gene sets which were generated and analysed (Figure 1. step 3).
(XLSX)

Table S3 **Cross-validation and details of classifier evaluation.** In the upper panel of this table the results for 10-fold cross-validation are provided for individual machine learning tools on the Wright et al. data. In the lower panels the results are shown for all individual classifiers, and meta-classifiers on data sets GSE32918, and GSE10846 divided into CHOP and R-CHOP components. For each component of the table classifiers are ordered by their average rank for survival separation (ABC vs GCB). Shown are the classifier identity, the Hazard Ratio (GCB vs ABC as baseline), 95% confidence interval of the hazard ratio, p-value and log rank.
(XLSX)

Table S4 **Overall classifier ranking and Type-III/unclassified assignments by top 3 classifiers.** The upper panel shows a summary of the performance of individual and meta-classifiers. Classifiers are order by their average rank for survival separation between ABC and GCB classes across the datasets GSE32918, and GSE10846 divided into CHOP and R-CHOP components. In the lower panel the top three classifiers LMT_J48_RF100_SMO, RF100_SMO, and SMO are compared for assignments to each class GCB, TypeIII, and ABC as % of cases, and ranked by Average % TypeIII (lowest to highest).
(XLSX)

Table S5 **Detailed class assignments for all data sets.** This file includes tables for all data sets used in this manuscript. It provides the available details for each sample (as indicated) such as GEO_Number, Original order, GEO_Class, Age, Sex and meta-data, followed by the classes assigned for each sample for every classifier tested including the selected class, the confidence of selected class, and the scores assigned for other classes. For data sets used in classifier training and development the results for all tested classifiers, and all combinations of classifier genes and training data sets are provided. For data sets used in later stages only classes assigned by DAC (LMT_J48_RF100_SMO) trained on Wright et al. data are shown.
(XLSX)

Table S6 **Confidence, threshold and survival.** Shown are the results observed with DAC (LMT_J48_RF100_SMO) using either the highest confidence for class-assignment or a hard threshold of confidence starting from p-value of 0.5 and incrementing in 0.1 steps. Shown are the Dataset identifier, the threshold used (PValCutoff), the resulting % Type-III/unclassified (%Unclassified), and the associated Hazard Ratios, and 95% Confidence Intervals of the Hazard Ratios for GCB vs ABC as baseline.
(XLSX)

Table S7 **ABC/GCB DLBCL Meta-profiles.** Shown are the complete lists of genes differentially expressed between ABC and GCB classes across all data sets. The table is divided into ABC and GCB class-associated genes. Shown are the Official Gene Symbol (Gene), the Median normalised fold change (MedianNormFC), the number of data sets (files) in which a gene was differentially expressed (Numfiles), the % of data sets in which the gene was differentially expressed (%GenePresent), followed by the Fold change in expression for each data set individually (GSE10846 divided into CHOP and R-CHOP components). A threshold of ≥6 data sets (NumFiles) was used for inclusion in meta-profiles.
(XLSX)

Table S8 **Meta-profile signature enrichment analysis.** The table is divided into two parts showing results for the ABC and GCB DLBCL meta-profiles separately. The first worksheet shows results for the ABC class (upABC_GCB), the second the results for the GCB class (ABC_upGCB). Shown are the enrichments observed for all signatures evaluated using the GeneSigDB, MSigDB and SignatureDB compendia as well as selected literature sources. For each signature the following are shown: the Gene Signature designation (Gene Signature), source of the signature (Source), number of genes overlapping between meta-profile and gene signature (Overlapping), number of genes in the signature (GeneSigSize), the average number of overlapping genes observed in 10^6^ random samplings (randomAvg), the standard deviation of observed for 10^6^ random samplings (randomAvg), the percentage of the gene signature that overlaps (%Overlap), whether a gene signature is enriched (Enriched) shown as 1 if probability of enrichment is <probability of depletion, the standard score for the observed overlap (ZScore), the probability of the overlap given the GeneSigSize (probablity), the Benjamini and Hochberg correction of probability scores (FDR), the enrichment or depletion of signatures and reciprocal signature pairs in ABC and GCB (Annotation), the genes contributing to overlap (EnrichedGenes), genes contributing to enrichment separated with ‘|’ (classifier genes excluded from analysis are located after ‘EXCLUDED_GENES:’ tag). For the “Annotation” column in each case the first symbol refers to the signature status in ABC (“+” = enriched (+ve Zscore), “−” = depleted (-ve Zscore), “*” = not significantly enriched or depleted) and the second symbol to the status in GCB. Where the signature is part of a signature pair (ie there are reciprocal signatures of up (_UP) and down (_DN) regulated genes for a given condition) these are separated by a “/”. These symbols can be used to search for signatures and signature pairs with coherent regulation.
(XLSX)

Table S9 **Signature enrichments for individual data sets.** The table is divided into two parts including results for all tested signatures for each data set individually in the comparison of ABC vs GCB DLBCL class. The first worksheet shows results for the ABC class (upABC_GCB), the second the results for the GCB class (ABC_upGCB). This emphasises the consistency of signature enrichment rather than the consistant differential expression of individual genes. For each signature the following are shown: the Gene Signature designation (Gene Signature), Source of the signature (Source), the number of data sets in which the signature was enriched/depleted (Number_of_Samples), the average Z score of the observed enrichment (avgZscore), the standard deviation of the Z scores of enrichment (sdZscore), the average probability (avgProb), the standard deviation of probabilities of enrichment (sdProb), whether there is consistent enrichment or depletion across data sets in which enrichment/depletion is observed (ZscoresAllSameSign), annotation of the signature and any associated reciprocal signature/signature pairs for each data set (shown using the data set identifier). For the latter in each case the first symbol refers to the signature status in ABC (“+” = enriched (+ve Zscore), “−” = depleted (-ve Zscore), “*” = not significantly enriched or depleted) and the second symbol to the status in GCB. Where the signature is part of a signature pair (ie there are reciprocal signatures of up (_UP) and down (_DN) regulated genes for a given condition) these are separated by a “/”. These symbols can be used to search for signatures and signature pairs with coherent regulation.
(XLSX)
