# Supplementary material for: A Microarray Platform-Independent Classification Tool for Cell of Origin Class Allows Comparative Analysis of Gene Expression in Diffuse Large B-cell Lymphoma
Source: PLoS One. 2013 Feb 12;8(2):e55895. doi: 10.1371/journal.pone.0055895 (PMC3570548; doi:10.1371/journal.pone.0055895)
Supplement: Figure S1 — Consistent classification and classification confidence. This accompanies Figure 2. The classes assigned by 31 tested classifiers for the (A) GSE10846 CHOP, (B) GSE10846 R-CHOP, (C) GSE32918 data set are shown along with published classes in GEO and those assigned by the LPS classifier (GCB = blue, Type-III = green, and ABC = yellow). Part (A) reproduces the data shown in Figure 2 and is included for completeness and to allow direct comparisons. As in Figure 2, samples are vertically ordered by the class given by the meta-classifier LMT_J48_RF100_SMO (later referred to as “DAC”); this meta-classifier assigns confidence scores for each class, and the class with highest confidence is selected for each sample. Within each class samples are ranked by classification confidence. At either extreme, samples are ordered from high to low confidence GCB, and from low to high confidence ABC. In the Type-III category high confidence cases are shown centrally flanked by lower confidence Type-III cases. On either side the latter are ordered by GCB or ABC signal (identified by GCB or ABC being the second highest classification confidence). The first column (labelled with black bar and red 5) identifies the classes assigned by LMT_J48_RF100_SMO, followed by results obtained for 30 other machine-learning classifiers, with the classes assigned for each case in the appropriate color. Classifiers are ranked (number above each column) from left to right according to the significance of survival separation between assigned ABC and GCB classes; note that LMT_J48_RF100_SMO was selected as the reference based on overall performance across multiple data sets, and in this data set is ranked 5th (shown in red) for survival separation. On the far right the published class assignments linked in GEO to the data set (GEO class, orange bar) and classes assigned by the LPS classifier using either a 0.8 or 0.9 p-value threshold classes are shown (dark gray bars respectively). (PDF) [file pone.0055895.s001.pdf]

A

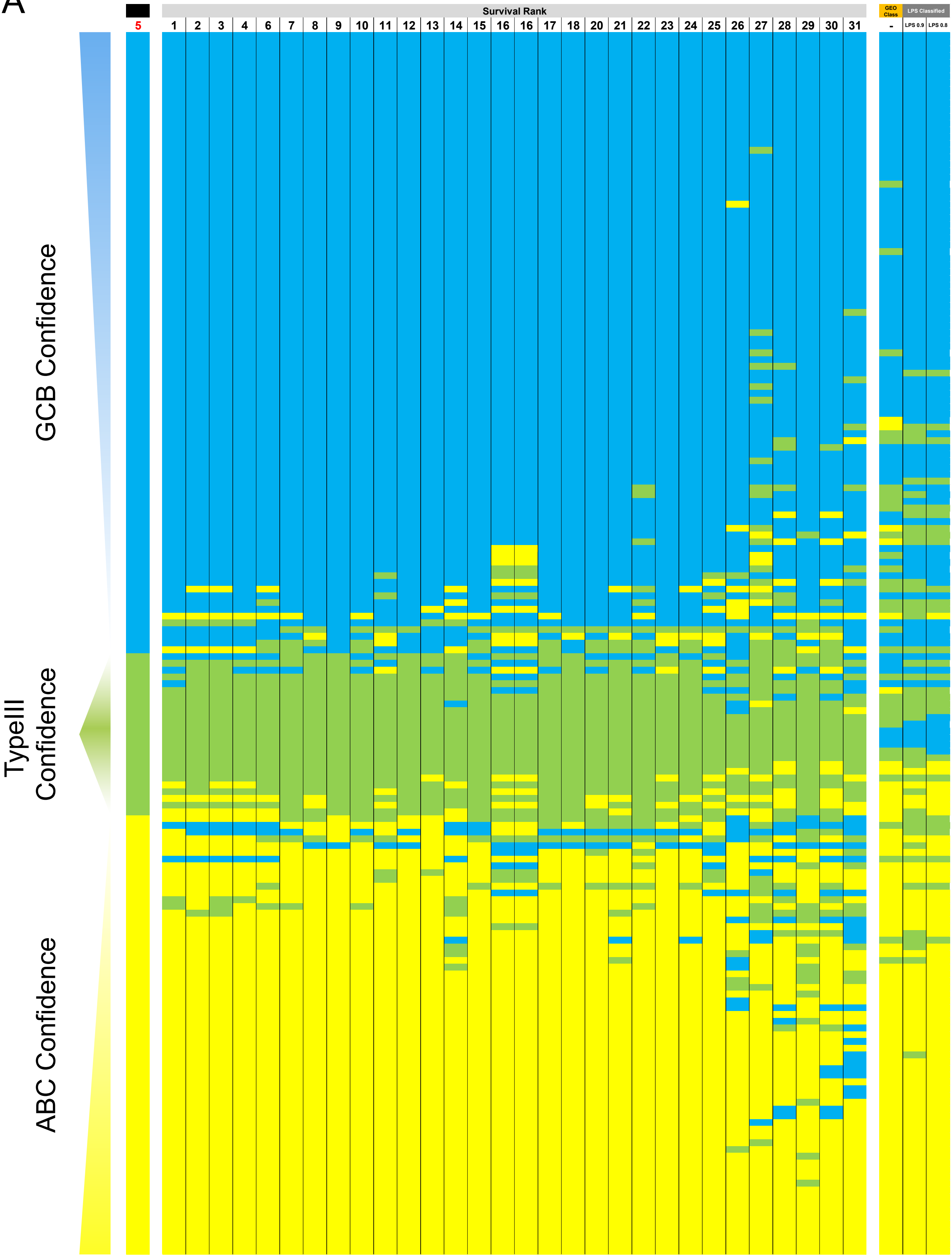

Survival Rank
5
1 2 3 4 6 7 8 9 10 11 12 13 14 15 16 16 17 18 20 21 22 23 24 25 26 27 28 29 30 31
GEO Class
-
LPS Classified
LPS 0.9
LPS 0.8
GCB Confidence
TypeIII Confidence
ABC Confidence

B

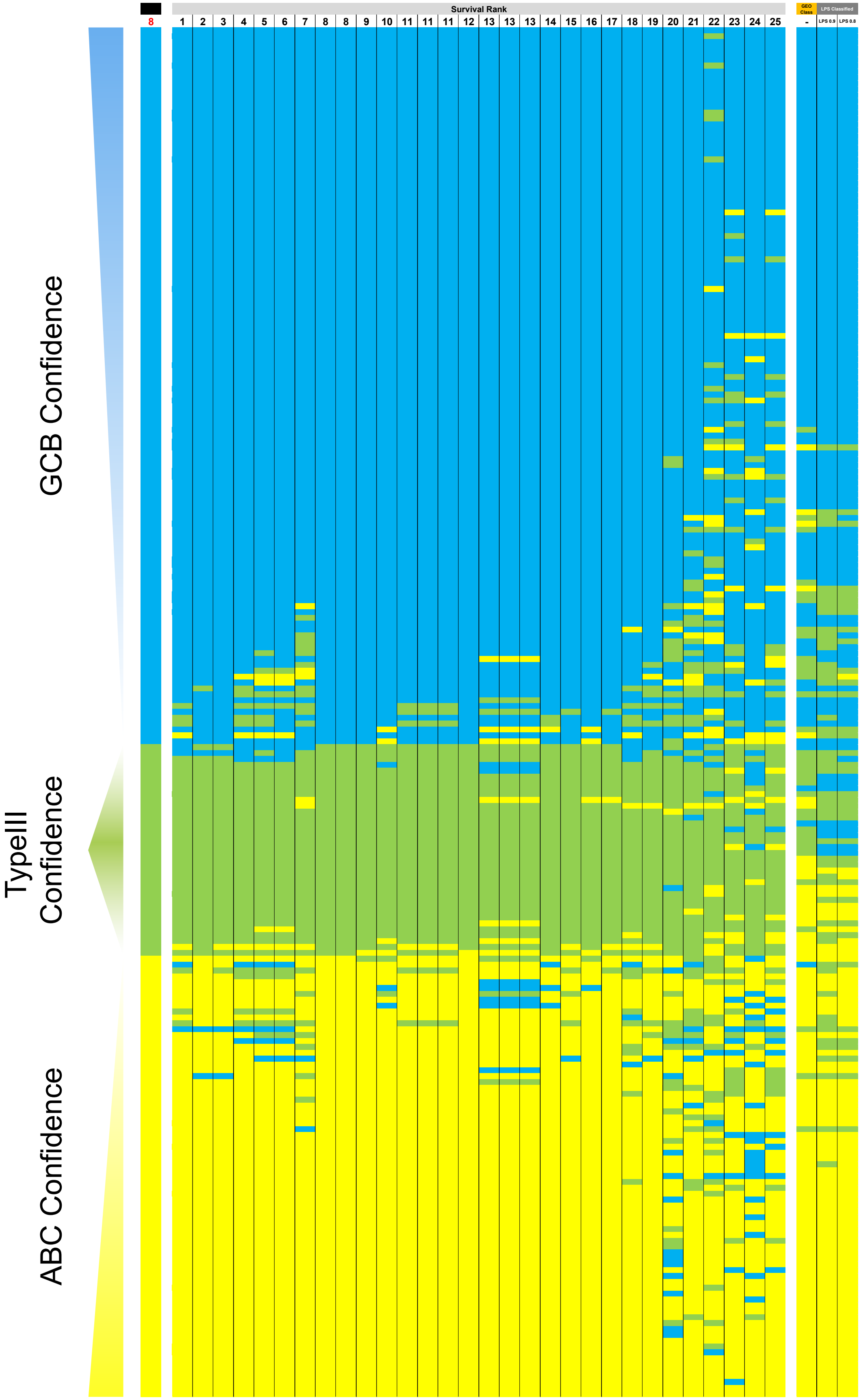

Survival Rank
8
1 2 3 4 5 6 7 8 8 9 10 11 11 11 12 13 13 13 14 15 16 17 18 19 20 21 22 23 24 25
GEO Class
LPS Classified
-
LPS 0.9
LPS 0.8
GCB Confidence
TypeIII Confidence
ABC Confidence

C

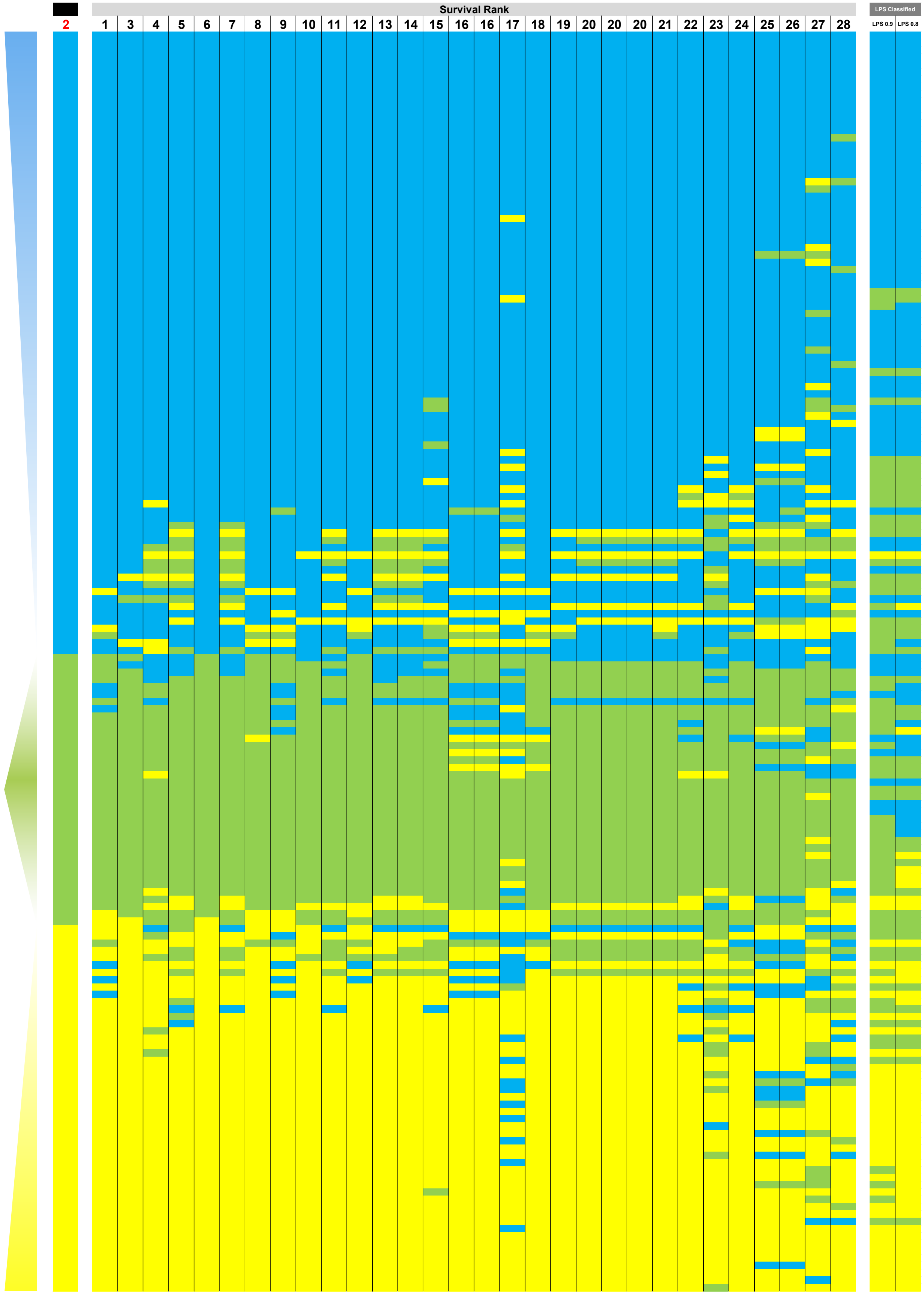

GCB Confidence
TypeIII Confidence
ABC Confidence
2
Survival Rank
1 3 4 5 6 7 8 9 10 11 12 13 14 15 16 16 17 18 19 20 20 20 21 22 23 24 25 26 27 28
LPS Classified
LPS 0.9
LPS 0.8
